# cGAS–STING, an important signaling pathway in diseases and their therapy

**DOI:** 10.1002/mco2.511

**Published:** 2024-03-23

**Authors:** Qijie Li, Ping Wu, Qiujing Du, Ullah Hanif, Hongbo Hu, Ka Li

**Affiliations:** ^1^ Sichuan province Medical and Engineering Interdisciplinary Research Center of Nursing & Materials/Nursing Key Laboratory of Sichuan Province West China Hospital, Sichuan University/West China School of Nursing Sichuan University Chengdu Sichuan China; ^2^ Department of Occupational Diseases The Second Affiliated Hospital of Chengdu Medical College (China National Nuclear Corporation 416 Hospital) Chengdu Sichuan China; ^3^ Center for Immunology and Hematology State Key Laboratory of Biotherapy West China Hospital, Sichuan University Chengdu Sichuan China

**Keywords:** agonist, cancer immunotherapy, cGAS–STING, inflammatory disease, inhibitor, signal transduction regulation, triggered DNA

## Abstract

Since cyclic guanosine monophosphate‐adenosine monophosphate synthase (cGAS)–stimulator of interferon genes (STING) signaling pathway was discovered in 2013, great progress has been made to elucidate the origin, function, and regulating mechanism of cGAS–STING signaling pathway in the past decade. Meanwhile, the triggering and transduction mechanisms have been continuously illuminated. cGAS–STING plays a key role in human diseases, particularly DNA‐triggered inflammatory diseases, making it a potentially effective therapeutic target for inflammation‐related diseases. Here, we aim to summarize the ancient origin of the cGAS–STING defense mechanism, as well as the triggers, transduction, and regulating mechanisms of the cGAS–STING. We will also focus on the important roles of cGAS–STING signal under pathological conditions, such as infections, cancers, autoimmune diseases, neurological diseases, and visceral inflammations, and review the progress in drug development targeting cGAS–STING signaling pathway. The main directions and potential obstacles in the regulating mechanism research and therapeutic drug development of the cGAS–STING signaling pathway for inflammatory diseases and cancers will be discussed. These research advancements expand our understanding of cGAS–STING, provide a theoretical basis for further exploration of the roles of cGAS–STING in diseases, and open up new strategies for targeting cGAS–STING as a promising therapeutic intervention in multiple diseases.

## Graphical abstract

cGAS–STING signaling pathway was discovered in 2013. With so many years of in‐depth research on cGAS–STING, people have realized that cGAS–STING plays a key role in human diseases, particularly DNA‐triggered inflammatory diseases and cancers. This review summarize the ancient origin, triggers, transduction, and regulating mechanisms of the cGAS–STING. The main directions and potential obstacles in the regulating mechanism research and therapeutic drug development of the cGAS–STING signaling pathway for inflammatory diseases and cancers will be discussed.

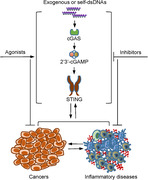

## 1 INTRODUCTION

Cyclic guanosine monophosphate‐adenosine monophosphate synthase (cGAS)–stimulator of interferon genes (STING) signaling pathway is the main mediator of DNA‐triggered immune response in mammalian cytoplasm. cGAS mainly recognizes and binds double‐stranded deoxyribonucleic acid (dsDNA) from pathogens or humans themselves. Activated cGAS catalyzes adenosine triphosphate (ATP) and guanosine triphosphate (GTP) to produce the second messenger cyclic guanosine adenosine monophosphate (cGAMP), which binds and activates STING on the endoplasmic reticulum (ER). Subsequently, STING‐dependent innate immune responses are activated.^1^, ^2^, ^3^, ^4^

The cytoplasmic DNA sensor cGAS was initially identified by Sun and Wu in 2013 and was shown to trigger IFN‐β in a manner that is dependent on STING.^5^ Since then, intensive studies have confirmed that cGAS–STING plays a significant role not only in pathogenic microbial infection but also in a variety of DNA‐triggered inflammation‐related diseases such as cancer, autoimmune diseases, neurological and visceral inflammation, which makes cGAS–STING signaling pathway a potentially effective therapeutic target for inflammatory diseases.^6^, ^7^, ^8^ Understanding the origin of the defense function of the cGAS–STING signal is helpful to understand better the triggering, transduction, and modifying process and molecular mechanism of human cGAS–STING signal, so as to further reveal the regulating mechanism of cGAS–STING signaling pathway in inflammatory diseases and cancers. Moreover, understanding the function and regulating mechanism of the cGAS–STING signaling pathway under different pathological conditions is of great significance for revealing the basic principles of cellular and molecular biology and studying the pathogenesis and therapeutic targets of human inflammatory diseases.

Here, we will summarize the ancient origin of the defense and transduction mechanism of the cGAS–STING signaling pathway, as well as the triggers from pathogens or humans themselves, the specific process and molecular mechanism of transduction after triggered, and the modification and regulation for the transduction process of cGAS–STING signal. We will also focus on the important regulating roles of the cGAS–STING signaling pathway under pathological conditions, such as infections, cancers, autoimmune diseases, neurological diseases, and visceral inflammations, and review the progress in drug development targeting the cGAS–STING signaling pathway. The main directions and potential obstacles in the regulating mechanism research and therapeutic agents development targeting the cGAS–STING signaling pathway in inflammatory diseases and cancers will be discussed, which will provide a theoretical and practical basis for further study on the precise triggers and regulating mechanisms of cGAS–STING signaling pathway in physiological and pathological conditions, and developing effective drugs with fewer side effects targeting cGAS–STING signaling pathway for the inflammation‐related diseases treatment, especially cancers.

## 2 EVOLUTION OF CGAS–STING SIGNAL

**FIGURE 1:**
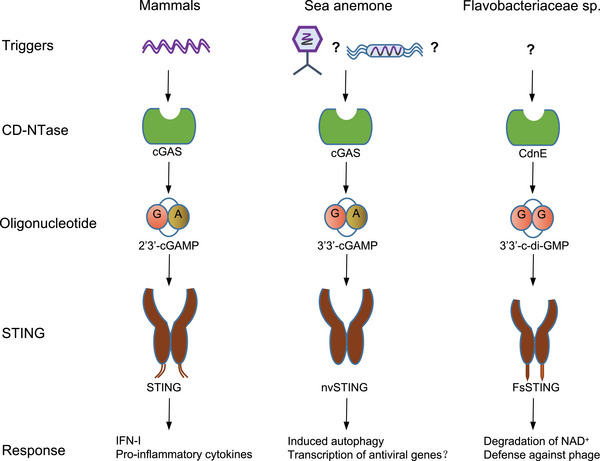
Signal transduction and evolutionary origin of CD‐NTase‐STING. In mammals, the cyclic guanylate adenylate synthase (cGAS)/DNCV‐like nucleotidyltransferase (CD‐NTase) family is activated by pathogens or self‐dsDNA to catalyze the production of 2′,3′‐cGAMP, activating stimulator of interferon genes (STING) to initiate the transcription of IFN‐I and proinflammatory cytokines. In the sea anemone *Nematostella vectensis*, the cGAS–STING signal is also present, and activated cGAS catalyzes the production of 3′,3′‐cGAMP, while the activation mechanism remains unclear. Although nvSTING can induce autophagy, it is unclear whether nvSTING induces gene transcription. In bacteria *Flavobactereae sp*., CdnE, a member of the CD‐NTase family, is activated by an unknown mechanism in response to phage infection and produces the second messenger 3′,3′‐c‐di‐GMP, thereby activating the antiviral reaction. Bacterial FsSTING contains a TIR domain that mediates NAD^+^ degradation upon activation. dsDNA, double‐stranded deoxyribonucleic acid; cGAMP, cyclic GMP‐AMP; c‐di‐GMP, cyclic guanosine diphosphate; nvSTING, *Nematostella vectensis* STING; FsSTING, *Flavobacterium sp*. STING; NAD^+^, nicotinamide adenine dinucleotide.

The cGAS–STING signal is a transmission mechanism with an ancient origin, and its existence in animals is earlier than that of interferon (IFN). The sea anemone *Nematostella vectensis* and mammals had a common ancestor more than 600 million years ago. Both species’ ancestor had cGAS and a binding STING molecule, and cGAS recognized cytoplasmic DNA and caused the formation of cyclic dinucleotides (CDNs), demonstrating the ancient and conserved function of the cGAS–STING signal.^1^, ^9^, ^10^, ^11^, ^12^, ^13^ In addition, the human cGAS–STING signal originates from the ancient mechanism of bacteriophage defense in bacteria (Figure 1).

### 2.1 cGAS–STING signal in metazoans

The findings show that a multitude of animals, from coral to mammals, encode cGAS‐like signal proteins, which produce an array of nucleotide second messenger signals.^14^ Bioinformatics analysis revealed that STING homologs existed in most animal genomes. Notably, STING from mammals and the sea anemone *Nematostella vectensis* (Cnidaria) can all bind CDN and have a similar reaction process.^10^

In mammals, the presence of DNA in the cytoplasmic matrix is sensed by cGAS, which triggers the production of CDN, including endogenous 2′,3′‐cGAMP. The carboxyl‐terminal domain of STING forms a homodimer to produce a concave binding pocket, which can bind with endogenous 2′,3′‐cGAMP or 3′,3′‐CDN released by prokaryotic pathogens to activate STING. Then, STING recruits downstream TANK‐binding kinase 1 (TBK1) to phosphorylate IFN regulatory factor 3 (IRF3), resulting in gene transcription of type I and III IFN (IFN‐I/IFN‐III) and other coregulatory factors.^10^, ^15^, ^16^ However, endogenous 2′,3′‐cGAMP is an agonist of human STING (hSTING), which is significantly more effective than prokaryotic 3′,3′‐CDN.^5^

The C‐terminal CDN receptor domain of *Nematostella vectensis* STING (nvSTING) has structural homology with hSTING. Moreover, the functional domain of STING and its CDN binding function are deeply conserved in the metazoan.^1^, ^17^ The structure prediction shows that the CDN synthetase in sea anemones has the same catalytic activity site as human cGAS and induces a strong hSTING‐dependent IFN signal in human cells.^1^ Therefore, cGAS and STING are evolutionarily conserved signal transduction components in animal biology (Figure 1). Human cGAS catalyzes the production of the second messenger cGAMP, which contains mixed 2′−5′ and 3′−5′ phosphodiester bonds (2′,3′), while the CDN synthase in sea anemone produces 3′,3′‐CDN different from human 2′,3′‐cGAMP, indicating that the mixed phosphodiester bonds found in human 2′,3′‐cGAMP are the adaptive evolution of the second messenger. When binding with STING, the unique structure formed by 2′,3′‐cGAMP due to mixed phosphate diester bonds is better than that formed by 3′,3′‐CDN, thus better binding to the evolutionarily conserved domain of STING.^1^, ^5^ Therefore, the cGAS–STING signaling pathway utilizes a unique evolutionary model, in which the protein components remain conservative in structure and the chemical bond changes of the second messenger promote functional innovation. In summary, the combination of STING with CDN is a long‐term evolutionarily and conserved function of STING. The cGAS–STING signal transduction has an ancient evolutionary origin, and the second messenger CDN in animals is earlier than the emergence of IFN signal transduction and modern innate immunity.

### 2.2 cGAS–STING signal originated in bacteria

In bacteria, cGAS/DncV‐like nucleotide‐transferase (CD‐NTase) catalyzes the production of a series of CDNs and trinucleotides (CTNs) to regulate various cell life activities, such as growth, osmoregulation, and chemotaxis.^18^ Interestingly, the four operons of the bacterial DncV gene, encoded CD‐NTase, are often clustered with the genes of the antiphage defense system. This defense was lost when two key amino acids in the enzyme's catalytic center were mutated, suggesting that the bacterial antiphage reaction requires the activity of CD‐NTase.^19^, ^20^ More interestingly, bioinformatics analysis revealed that some genes in the operons of phage defense genes encoded the proteins homologous to STING.^16^, ^21^ Although the overall structure of the metazoan STING is conserved, the bacterial STING protein is smaller and very compact. For example, *Flavobactereae sp*. STING (FsSTING) (IMG Gene ID 2624319773) shows a standard V‐shaped homodimer structure and a hydrophobic α helix, similar to the STING protein structure of metazoans.^1^, ^5^, ^17^, ^22^ Bacterial STING is bound to cyclic guanosine diphosphate (c‐di‐GMP), while it is poorly or not bound to CDNs containing 2′−5′ bonds such as 2′,3′‐cGAMP. Structural prediction shows that most bacterial STING proteins contain Toll/interleukin‐1 (IL‐1) receptor (TIR) domains without transmembrane (TM) helices.^23^ In fact, c‐di‐GMP activates the bacterial TIR‐STING protein to lead to rapid and efficient hydrolysis of nicotinamide adenine dinucleotide (NAD), accompanying by TIR‐STING being assembled into filaments, similar to the oligomerization when STING binds 2′,3′‐cGAMP to activate TBK1 (Figure 1).^16^, ^24^ The cyclic oligonucleotide‐based antiphage signaling system (CBASS) in bacteria is similar to the cGAS‐dependent immune response system that senses virus replication in human cells. CBASS depends on activating the CD‐Ntase enzyme to initiate the second messenger‐dependent antiviral response.^2^, ^19^, ^25^, ^26^, ^27^ Thus, CBASS plays a role in controlling bacteriophage infection in bacteria, indicating that the antiviral function of cGAS in vertebrates may originate from prokaryotes.^19^

In conclusion, prokaryotic cells already contain and are active in the CD‐NTase enzyme, which generates CDN signals, and the STING‐like molecule, which serves as the signal receptor. Crucially, both of these molecules have roles in viral defense. However, the human cGAS–STING signal can be activated not only by viruses but also by multiple triggers.

## 3 cGAS–STING TRIGGERS

**FIGURE 2:**
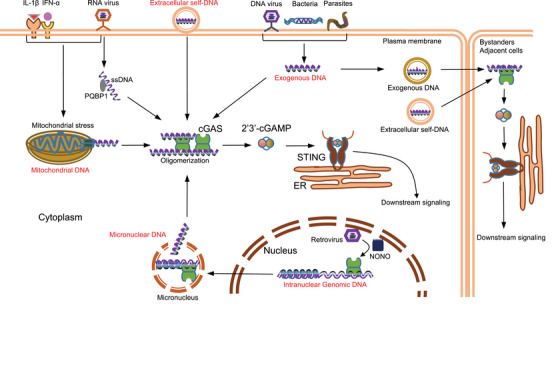
Triggers of cGAS–STING signaling pathway. The cGAS–STING signal can be activated by exogenous DNA, extracellular self‐DNA, mitochondrial DNA, intranuclear genomic DNA, and micronucleus DNA (red font). Exogenous DNA is mainly derived from DNA viruses, bacteria, and parasites, which can also be encapsulated into vesicles to trigger cGAS in bystanders and adjacent cells via paracrine diffusion. In addition, retroviruses, with the assistance of NONO, bring cGAS to the site that HIV integrates and replicates in the nucleus, resulting in cGAS sensing HIV reverse‐transcribed DNA in the nucleus. PQBP1 promotes the interaction of ssDNA from RNA virus reverse transcription with cGAS to activate STING signal efficiently. Extracellular self‐DNA: increased nonapoptotic cell death, such as tumor cell necrosis, promotes its DNA to be encapsulated into vesicles into bystanders or adjacent cells. Mitochondrial DNA: RNA viruses, IL‐1β, and IFN‐α induce mitochondrial stress, resulting in the exposure of mitochondrial DNA into the cytoplasm to be sensed by cGAS. Intranuclear genomic DNA: when organisms are exposed to environmental stimuli such as reactive oxygen species (ROS) or ultraviolet (UV) radiation, genomic DNA in the nucleus may be damaged, and the leakage of damaged DNA into the cytoplasm triggers the activation of cGAS. During mitosis in proliferating cells, cGAS enters the nucleus and binds to chromatin DNA. However, the interaction of cGAS with chromatin is not or is not only mediated by DNA but may also involve protein‐protein interactions, which may be the key to preventing GAS activation. Micronucleus DNA: irreversible mitotic DNA damage, chromosome missegregation, or other DNA replication problems usually result in the formation of a micronucleus at the end of mitosis. However, the micronucleus membrane is fragile and easily ruptured, resulting in the exposure of micronucleus DNA to the cytoplasm. NONO, non‐POU domain‐containing octamer binding protein; PQBP1, polyglutamine binding protein 1; IL‐1β, interleukin‐1β.

Human cGAS–STING originates from the ancient mechanism of bacteriophage defense in bacteria. In fact, its important and conservative function is still to recognize triggers and mediate immune defense. Briefly, cGAS senses DNAs from the host self and microorganisms to trigger the STING‐dependent innate immune response, preventing host from infection or maintaining self‐cells' stability. Exogenous DNAs from microorganisms are still the main factors to activate the cGAS–STING signal, including DNAs from viruses, bacteria, and parasites. In addition, cGAS senses various types of endogenous DNAs, including extracellular self‐DNA, mitochondrial DNA, intranuclear genomic DNA, and micronucleus DNA. Here, we summarize the different sources of DNAs that can activate cGAS and the processes involved (Figure 2).

### 3.1 Exogenous DNA

The exogenous DNAs of microorganisms are usually packaged into a nucleoid or tight protein–DNA complex structure, thus shielding them from entering the cytosol. However, the breakdown of microbial cell structure may cause its DNA to be exposed to the host cytoplasm, which is recognized by cGAS to activate the STING signaling pathway.

Herpes simplex virus type 1 (HSV‐1) was the first DNA virus to be shown to activate STING in vivo and in vitro, thus being widely used as a STING activator in laboratory experiments.^28^ Known Kaposi sarcoma‐associated herpesvirus (KSHV),^3^ human adenovirus (Ads),^29^ murine gamma‐herpesvirus 68 (MHV68),^30^ vaccinia virus,^31^ and cytomegalovirus (CMV)^32^, ^33^ can all activate the cGAS–STING signaling pathway in the host cytoplasm. In addition, reverse‐transcribed dsDNA from viral RNA of human immunodeficiency virus (HIV) can also induce the cGAS–STING signaling pathway, which is made possible by the abundant presence of cGAS in the host nucleus.^34^ During HIV‐2 infection, a cofactor called non‐POU domain‐containing octamer binding protein (NONO) binds to HIV nucleic acid and capsid proteins and brings cGAS to the site where HIV is integrating and replicating, causing cGAS to sense HIV reverse‐transcribed DNA in the nucleus.^35^ In addition, polyglutamine binding protein 1 (PQBP1), involved in RNA splicing, promotes the interaction between HIV‐1 reverse‐transcribed ssDNA and cGAS in dendritic cells (DCs), effectively activating the STING signaling pathway and producing IRF3 with the assistance of zinc finger CCHC‐type containing protein 3 (ZCCHC3) and GTP‐activated protein SH3 domain binding protein 1 (G3BP1).^36^, ^37^

cGAS can also sense intracellular bacterial DNAs,^10^ including *Mycobacterium tuberculosis*,^38^, ^39^, ^40^ *Chlamydia*,^41^ *Listeria monocytogenes*,^42^ *Francisella*,^43^ *Neisseria gonorrheae*,^44^ *Streptococcus pneumoniae*,^45^ and *Streptococcus pyrogen*.^46^ However, some intracellular bacteria, such as *Staphylococcus aureus* ^47^ and *Vibrio cholerae*,^48^, ^49^ can produce CDN to bypass cGAS and directly activate STING. In addition, cGAS can also sense the genomic DNA of protozoa such as *Plasmodium* and *Toxoplasma gondii*, which is conducive to activating the host immune response to control the proliferation of parasites.^50^, ^51^

In addition to triggering cGAS in the host cytoplasm, exogenous microbial DNAs are wrapped in extracellular vesicles to trigger cGAS of bystanders and adjacent cells via paracrine diffusion. However, this paracrine cGAS–STING signal transduction occurs between cells infected by intracellular bacteria and activated T cells, leading to increased T cell apoptosis and impaired antibacterial defense.^52^

### 3.2 Self‐DNA

Normally, the DNAs of human self‐cells are not exposed too much to trigger cGAS, because there are many active enzymes in human cells to cut and clear away unwanted DNAs, thus avoiding inducing autoimmunity. Here, we briefly summarize these exoenzymes and their functional characteristics.

Three‐prime repair exonuclease (TREX1) is a cytosolic exonuclease that eliminates both unmodified and oxidized cytoplasmic DNA.^53^ Extracellular and lysosomal endonucleases are responsible for eliminating exogenous and self‐DNA from dead cells.^54^ Ribonuclease H2 (RNaseH2) is a ribozyme that excises the ribonucleotides wrongly mixed into genomic DNA.^55^ SAM and HD domain‐containing deoxynucleoside triphosphate triphosphohydrolase 1 (SAMHD1) can degrade all four deoxynucleoside triphosphates, thus coordinating their intracellular levels and genomic stability.^56^, ^57^ However, cGAS can sense the DNAs from multiple sequences and cell types. Therefore, when the above preventive functions are dysfunctional or the above enzymes make mistakes, the cGAS–STING signaling pathway is strongly activated by self‐DNA, ultimately leading to autoimmunity. Here, we review the types of DNAs that can trigger the cGAS–STING signaling pathway and the reaction processes.

#### 3.2.1 Extracellular self DNA

With the increase of nonapoptotic cell death, once phagocytosis and digestion are damaged or extracellular and lysosomal DNase are defective, self‐DNAs are exposed into the cytoplasm to activate cGAS–STING signal. For example, during radiotherapy for cancer patients, a large amount of tumor‐derived DNAs are delivered from the extracellular space into DCs by direct endocytosis of exosomes, activating the cGAS–STING signaling pathway and increasing antitumor immunity.^58^, ^59^, ^60^ Moreover, acute ischemia, such as coronary artery occlusion, causes many cells to die, leading monocytes to recruit and engulf the dead cell DNAs, thus activating the STING‐dependent inflammatory reaction.^61^ In addition, in aseptic inflammatory diseases, the formation of neutrophil extracellular traps (NETs) leads to cell death and the leakage of neutrophil DNA–protein complexes into the extracellular space, thus activating the cGAS–STING signaling pathway.^62^ Extracellular DNA is the main factor to induce many autoimmune inflammatory reactions, but the molecular mechanism needs to be fully clarified.

#### 3.2.2 Mitochondrial DNA

The release of mitochondrial DNA (mtDNA) into the cytoplasmic matrix has become the main trigger for activating the cGAS–STING signaling pathway.^63^ Normally, mtDNA is wrapped in two membrane systems that cGAS cannot access: the outer and inner mitochondrial membranes (OMM and IMM).^64^ However, mitochondrial integrity may be damaged after mitochondrial injury and cell apoptosis, resulting in the leakage of mtDNA into the cytosol and the release of cytochrome *C* into the cytoplasm matrix to trigger the canonical mitochondrial pathway of apoptosis.^8^, ^65^ The formation of BAX/BAK holes in the OMM causes the mitochondrial outer membrane to become permeable, thus allowing the IMM to extrude into the cytoplasm. Then, mtDNA leaks into the cytoplasm through the broken hole of the inner nuclear membrane,^8^, ^66^, ^67^ subsequently activating the cGAS–STING signaling pathway to produce IFN‐I. In addition, voltage‐dependent anion channels 1 and 3 (VDAC1 and VDAC3)^63^ induce the formation of holes in the OMM, leading to the leakage of mtDNA fragments into the cytoplasm. How mtDNA penetrates from IMM is still unclear, but it may be related to the mitochondrial permeability transition pore (MPTP), which spans the IMM and is triggered to assemble under various cell stresses.^68^, ^69^

Impaired replication and repair processes of mtDNA can lead to the activation of the cGAS–STING signal.^10^ For example, after the depletion of transcription factor A mitochondria (TFAM), mtDNA is encapsulated in a defective nucleoid structure to leak into the cytoplasm.^70^ Furthermore, mtDNA stress caused by the lack of mitochondrial endonuclease G or chemotherapy drugs can activate cGAS.^68^, ^71^ The mtDNA may be modified by mitochondrial reactive oxygen species (ROS), which can make mtDNA more resistant to the nuclease TREX1, thus more stimulating to the cGAS–STING signal.^71^ In addition, the cytokine signal cascade can also trigger mitochondrial dysfunction and associated mtDNA release, but the mechanism by which mtDNA is released from mitochondria under this condition remains unclear. For example, both inflammatory cytokines IL‐1β and IFN‐α can impair mitochondrial homeostasis and activate cGAS via mtDNA, triggering the amplification of the immune response.^72^ Through this active regulation, various cytokine signaling pathways may lead to increased inflammation mediated by cGAS recognition of mtDNA. Finally, pathogenic microorganisms can induce mitochondrial stress and lead to mtDNA leakage, thus indirectly triggering cGAS–STING signal activation.

In summary, mtDNA become a broad trigger factor of the immune response by cGAS–STING signal transduction, which is also the important cause of many autoimmune reactions.

#### 3.2.3 Intranuclear genomic DNA

The damage of genomic DNA in the nucleus causes its fragments to leak into the cytoplasm.^8^ For example, when the oncogene H‐RasV12 induces cell aging, the damage or replication failure of genomic DNA in the nucleus lead to the production of cytoplasmic chromatin fragments (CCFs), thus activating the cGAS–STING signaling pathway in the cytoplasm to cause transient or chronic inflammation^73^ and mediate IFN‐β production.^74^ In addition, the damage of nucleus genomic DNA often occur when an organism is exposed to environmental stimuli (such as ROS or ultraviolet [UV] radiation), also leading to the leakage of damaged DNA into the cytoplasm to trigger the activation of cGAS, thus showing increased oxidative damage of self‐DNA accompanied by increased IFN‐I levels.^8^

Whether genomic DNA in the nucleus directly triggers cGAS is unknown, while the possibility is increased by the localization of cGAS in the nucleus. It has been proven that aberrant cGAS activity can directly sense genomic DNA in the nucleus under some disease states.^65^ During mitosis in proliferating cells, cGAS enters the nucleus and binds to chromatin DNA.^75^ However, the interaction of cGAS with chromatin is not or is not only mediated by DNA but may also involve protein–protein interactions, which may be the key to preventing GAS activation.^76^, ^77^ Furthermore, in vitro experiments showed that cGAS was activated by chromatin or nucleosomes.^78^ However, other studies have reported that nucleosome core particles did not cause the activation of cGAS, although cGAS bound them with high affinity.^76^ The differences in these studies are unclear, but it can be determined that the activation of cGAS by is caused not by nucleosomes but by spliced DNA or residual free DNA. Therefore, the mechanism by which genomic DNA triggers cGAS in the nucleus remains to be fully clarified.

#### 3.2.4 Micronuclear DNA

Irreversible mitotic DNA damage, chromosome mis‐separation, and other DNA replication problems usually result in the formation of micronuclei at the end of mitosis,^79^ which contain chromatin fragments surrounded by nuclear membrane‐like structures or undisassembled mitotic chromosomes. However, a variety of “noncore” envelope proteins, including nuclear pore complex components, fail to assemble onto the chromosomes in the micronucleus, thus resulting in multiple “nuclear” processes not working properly in the micronucleus. Moreover, compared with the membrane structure of the main nucleus, the membrane of the micronucleus is more fragile. Thus, the membrane of the micronucleus is easily ruptured, causing chromatin to leak into the cytoplasm.^80^, ^81^, ^82^ In addition, the micronucleus contains heterochromatin (trimethylated histone H3: H3K9me3 and H3K27me3) and a DNA damage marker (γ‐H2AX), indicating that they come from the transcriptional silencing region of the genome,^73^, ^83^ which may be the reason why micronucleus chromatin effectively triggers cGAS. It has been shown that the inactivation of the RecQ‐like helicase BLM,^84^ RNase‐H2,^85^ tumor suppressor gene BRCA2,^86^ topoisomerase II,^87^ or poly ADP ribose polymerase 1 (PARP1) inhibitor^88^ lead to the inhibition of DNA replication, repair, and recombination, thus resulting in the increased micronuclei to activate the cGAS–STING signaling pathway. For example, the replication crisis of cell cycle‐arrested precancerous cells promote an increase in cGAS‐sensing micronuclei, and cGAS activation prevents further transformation of precancerous cells by inducing autophagy‐dependent cell death. Therefore, the precancerous and senescent cells or cells with mitotic disorders could be eliminated via the activation of the cGAS–STING signaling pathway.

## 4 cGAS–STING SIGNAL TRANSDUCTION MECHANISM

**FIGURE 3:**
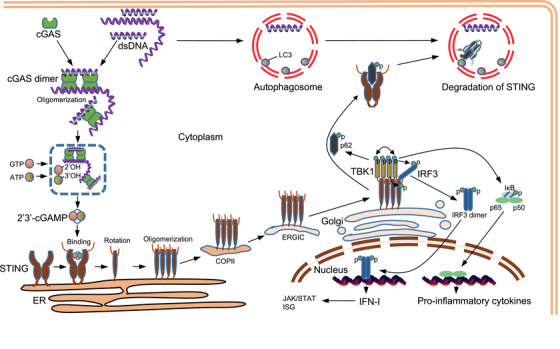
Signal transduction mechanism of cGAS–STING. The cytoplasmic dsDNA is sensed by cGAS, which is activated to form a dimer, assembling a higher‐order oligomer of cGAS:dsDNA (2n:2) with dsDNA. Subsequently, cGAS catalyzes GTP and ATP to generate 2′,3′‐cGAMP, which binds to STING in the endoplasmic reticulum (ER), inducing allosteric and rotation of STING to further form higher‐order oligomers. Oligomerized STING is transported from ER to Golgi by COPII and ERGIC vesicles to recruit multiple TBK1 molecules. Then, adjacent TBK1 molecules phosphorylate each other and cause phosphorylation of adjacent STING to form a platform for IRF3 recruitment. TBK1 binding to STING leads to the phosphorylation of IRF3, which then forms an IRF3 dimer and enters the nucleus to induce the transcription of IFN‐I. The binding of IFN‐I to its receptor further activates the JAK/STAT signaling pathway and induces the transcription of ISG. TBK1 also leads to the phosphorylation and degradation of IκB, and the released NFκB (p65/p50) enters the nucleus to induce the transcription of proinflammatory cytokines. In addition, dsDNA induces autophagosome formation and phosphorylation of p62 by activated TBK1 to promote its binding to STING and guide STING into autophagosomes for degradation. COPII, cytoplasmic coat protein complex II; ERGIC, ER‐Golgi intermediate compartment; IFN‐I, type I interferon; IκB, inhibitor of NF‐κB; IRF3, interferon regulatory factor 3; ISG, IFN‐stimulating gene; JAK/STAT, Janus kinase/signal transducer and activator of transcription; NF‐κB, nuclear factor kappa‐B; TBK1, TANK‐binding kinase 1.

As described above, a variety of DNA triggers can be recognized by and bound to cGAS. After undergoing a conformational change to the active state, cGAS catalyzes ATP and GTP to produce cGAMP, a second messenger that binds and activates STING on the ER. Activated STING is then transferred to the Golgi apparatus, where it activates TBK1, inducing TBK1‐dependent phosphorylation of itself (i.e., self‐phosphorylation), STING, and IRF3 (the downstream transcription factor). Finally, IRF3 phosphorylation triggers IRF3 dimerization, facilitating its entry into the nucleus to trigger the transcription of IFN‐I and proinflammatory cytokines (Figure 3).^6^, ^52^, ^89^

### 4.1 cGAS recognizes cytoplasmic DNA

cGAS is a member of the structurally conserved CD‐Ntases superfamily, which is ubiquitously expressed across prokaryotes and eukaryotes. cGAS is comprised of a disordered N‐terminal domain and a catalytic C‐terminal domain. The N‐terminal portion of the catalytic domain is comprised of two helices and a highly‐twisted β‐pleated sheet, and it includes all the catalytic residues (Glu225, Asp227, and Asp319). The C‐terminal portion of the catalytic domain is comprised of a helical beam containing a conserved zinc ion‐binding domain, which forms a surface groove at the back of the catalytic domain and is the main binding site for DNA. Upon binding, DNA stimulates cGAS activation by inducing conformational changes around the catalytic sites.^26^, ^89^ cGAS is only activated by type B dsDNA binding (it does not recognize type A dsDNA or RNA), and it demonstrates no dsDNA sequence specificity. Moreover, at least 20 bp of dsDNA are required for dimer formation (and full activation), with dsDNA fragments less than 20 bp inducing weaker cGAS dimer formation. Importantly, in vitro studies suggest that cGAS catalytic activity is only promoted when cGAS is present at very high concentrations, and dimer formation is not promoted at low cGAS concentrations.^90^, ^91^, ^92^, ^93^, ^94^ There are two main dsDNA‐binding sites on opposite sides of the catalytic domain of cGAS, the primary contact surface for dsDNA and a complementary dsDNA‐binding site. Thus, dsDNA binding yields a cGAS:dsDNA (2:2) complex structure. In addition, cGAS dimer formation aggregates two dsDNA molecules together, facilitating continuous recruitment of cGAS. Through this process, a higher‐order “ladder” oligomer of cGAS:dsDNA (2n:2) is formed.^52^, ^93^, ^95^ After activation via DNA binding‐induced structural changes, cGAS catalyzes its substrates GTP and ATP, transfers ATP to the 2′‐OH of GTP, and generates linear iso‐dinucleotide phosphate pppG (2′−5′) pA as the substrate. Then, cGAS transfers part of GTP to the 3′‐OH of adenosine phosphate through intramolecular transfer to produce 2′,3′‐cGAMP. cGAS also catalyzes the synthesis of linear dinucleotides, such as AMP‐2′‐ATP, GMP‐2′‐GTP, and AMP‐2′‐GTP.^96^, ^97^ This specific chemical bond is an adaptive evolution of metazoan, and it is conducive to generating metazoan‐specific signals to activate the immune response.

### 4.2 cGAMP binds and activates STING

The asymmetric 2′,3′‐cGAMP, the catalytic product of cGAS, has a high affinity for STING, and 2′,3′‐cGAMP binding promotes STING dimerization.^5^, ^34^ Here, the most important role of cGAMP is to bind and activate STING. STING is a small molecular weight protein (approximately 40 kDa) comprised of a short N‐terminal cytoplasmic segment, four TM helices that traverse on the ER membrane, a C‐terminal tail (CTT) responsible for binding TBK1, and a cytoplasmic ligand binding domain (LBD).^89^ When cGAMP binds to the V‐shaped dimer pocket formed by two STING protein cytoplasmic regions, the LBD of the STING dimer is converted into a four‐stranded β‐sheet “lid,” resulting in the closure of the ligand‐binding pocket, and inducing STING to activate downstream signaling. Specifically, cGAMP induces the opening of the connecting ring between the LBD and TM domains of the STING protein and then mediates the interactions between STING dimers, forming STING oligomers.^17^, ^98^ In humans, STING forms disulfide bonds at Cys148, which facilitates crosslinking of STING dimers and stabilization of higher‐order oligomers. In addition, STING is palmitoylated at Cys88 and Cys91, and this may also promote STING oligomerization.^99^, ^100^ The STING dimers are subsequently integrated into cytoplasmic coat protein complex II (COPII) vesicles and translocated from the ER to perinuclear ER‐Golgi intermediate compartment (ERGIC) vesicles for transport to the Golgi body. In addition to its role in the activation of STING, cGAMP acts as a second messenger. cGAMP is transferred between cells in a variety of ways, including connexin‐dependent intercellular transfer via gap junctions, by entry into bystander cells following cell fusion, and by inclusion in extracellular vesicles for extracellular remote transport.^12^, ^101^, ^102^, ^103^, ^104^, ^105^

### 4.3 STING recruites and activates TBK1, leading to phosphorylation and activation of IRF3

After transfer to the Golgi complex, STING dimers are known to recruit TBK1. This interaction between STING and TBK1 is mediated by an evolutionarily conserved motif composed of eight amino acid residues at the C‐terminal end of STING. The oligomerization of activated STING induces phosphorylation of the adjacent TBK1, which then itself phosphorylates adjacent STING molecule.^24^, ^106^ Specifically, the conserved TBK1 binding motif (TBM) in the unstructured STING CTT region becomes inserted into the groove formed by the kinase domain of TBK1 and the scaffold dimer domain (SDD).^24^, ^98^, ^106^ STING oligomerization and the interaction between STING oligomers and TBK1 result in the accumulation of multiple TBK1 molecules, further promoting the mutual phosphorylation of TBK1. Because the Ser366 phosphorylation site in the STING CTT cannot approach the kinase active site of the TBK1 molecule that directly binds it, Ser366 binds the kinase active site of a TBK1 that is bound to another STING and is subsequently phosphorylated.^24^, ^105^, ^107^ In addition, phosphorylated STING provides a docking site for an IRF3 monomer, which is recruited through interactions with its positively charged surface, bringing the IRF3 monomer close to catalytically active TBK1.^17^, ^106^, ^107^ To summarize, TBK1 phosphorylates the CTT region of a specific STING monomer (to which it is not bound) to recruit IRF3, and IRF3 is then efficiently phosphorylated by TBK1 bound to the same STING monomer. The phosphorylated IRF3 forms a dimer and enters the nucleus to promote IFN‐I transcription. Subsequently, IFN‐I is transported between cells in a paracrine or autocrine manner. After binding its receptors, IFN‐I triggers the Janus kinase/signal transducer and activator of transcription (JAK/STAT) signaling pathway, ultimately inducing transcription of the IFN‐stimulating gene (ISG).^89^, ^108^ In addition, because TBK1 can induce the phosphorylation of IκB, an inhibitor of NF‐κB, facilitating its degradation, TBK1 recruitment by STING facilitates NF‐κB transport to the nucleus where it triggers the production of proinflammatory cytokines.

### 4.4 Attenuation of cGAS–STING signal

After activating the cGAS–STING signal, cytoplasmic DNA induces the formation of double‐membrane autophagy, the reduced phosphorylation of STING by Unc‐51‐like kinase 1 (ULK1) at S555, the transformation of LC3, the turnover of ubiquitin‐binding autophagy receptor p62, and the combination of autophagy associated protein 3 (ATG3) to induce the degradation of STING.^109^, ^110^, ^111^ Interestingly, DNA‐stimulated degradation of STING is also dependent on TBK1. After activated TBK1 recruits and phosphorylates IRF3, p62 is recruited to TBK1 and phosphorylated, thus further enhancing the affinity of p62 for ubiquitinated STING and promoting its transport to autophagosomes. Through these mechanisms, DNA and cGAMP mediate the activation of the autophagy pathway.^112^ Importantly, a preprogrammed STING degradation mechanism ultimately weakens signal transduction, preventing excessive inflammation.^113^ Thus, Unc‐93 homolog B1 (UNC93B1) provides a crucial check on STING to prevent hyperactivation, attenuating the cGAS–STING signaling pathway by targeting STING for autophagy‐lysosome degradation.^114^

## 5 POSTTRANSLATIONAL MODIFICATION IN THE cGAS–STING SIGNALING PATHWAY

The cGAS–STING signaling pathway has a strict and complex signal transduction mechanism, so it must be strictly regulated to maintain the normalization of function.^115^ Posttranslational modification (PTM) plays an important role in regulating cGAS and STING. Here, we summarize the research progress on PTMs regulating cGAS/STING (Table 1).

**TABLE 1:** PTM of the cGAS/STING signaling pathway.

Target	Regulatory protein	PTM mode	Site; Mechanism	References
cGAS	B lymphoid tyrosine kinase	Phosphorylation	Y215; Maintaining its cytosolic localization	^116^
cGAS	Akt	Phosphorylation	S305 (mouse S291); Blocking the binding and catalysis of cGAS to ATP and GTP	^117^
cGAS	Protein phosphatase 6	Dephosphorylation	S435 (mouse S420); Enhancing the binding affinity and enzyme activity of cGAS‐GTP	^118^
cGAS	Cyclin‐dependent kinase 1 (CDK1)–cyclin B kinase complex	Phosphorylation	S305; Blocking the synthesis of cGAMP	^119^
cGAS	Protein phosphatase 1 (PP1)	Dephosphorylation	S305; Removal of phosphorylation of cyclin‐dependent kinase 1 (CDK1)–cyclin B kinase complex	^119^
cGAS	Aurora B	Phosphorylation	Ser13, S37, S64, T69, T91, S116, S129, and S143; Blocking the recognition of chromatin DNA	^120^
cGAS	Protein phosphatase 1(PP1) or protein phosphatase 2A	Dephosphorylation	Removal of phosphorylation of Aurora B	^120^
cGAS	TRIM56	Ubiquitylation	K335; Enhancing dimerization of cGAS	^123^
cGAS	RNF185	Ubiquitylation	K137/384; Enhancing enzyme activity	^152^
cGAS	USP14	Deubiquitination	Inhibition of p62‐dependent degradation of cGAS	^115^, ^125^
cGAS	USP27X/USP29	Deubiquitination	Promote the stability of cGAS	^126^, ^127^
cGAS	SENP7	Desumoylation	K335, K372, and K382; Promoting DNA binding, oligomerization, and nucleotide transferase activity of cGAS	^129^
cGAS	TRIM38	Sumoylation	K464, K217; Preventing K48‐linked polyubiquitination and subsequent cGAS proteasome degradation	^129^
cGAS	SENP2	Sumoylation	Promoting polyubiquitination of cGAS and subsequent proteasomal degradation	^129^
cGAS	TTLL6	Glutamylation	E272; Inhibiting the DNA‐binding capacity of cGAS	^115^, ^130^
cGAS	TTLL4	Glutamylation	E302; Inhibiting the enzymatic activity of cGAS	^115^, ^130^
cGAS	CCP6/CCP5	Deglutamylation	Restoring the DNA binding and catalytic activity of cGAS	^130^
cGAS	Caspase 1	Proteolysis	D140/D157; Preventing the activation of cGAS	^132^
cGAS	Caspase 1	Proteolysis	D319; Preventing the activation of cGAS	^135^
cGAS	Aspirin	Acetylation	K384, K394, and K414; Preventing the activation of cGAS	^136^
cGAS	Lysine acetyltransferase 5	Acetylation	K47, K56, K62, and K83; Promoting the DNA‐binding ability of cGAS	^137^
cGAS	Protein arginine methyltransferase 5	Methylation	R124; Blocking cGAS from binding to DNA	^138^
STING	RNF5	Ubiquitylation	K150; Promoting the degradation of STING	^140^
STING	TRIM29	Ubiquitylation	K288, K337; Promoting the degradation of STING	^141^
STING	TRIM30α	Ubiquitylation	Mouse K275; Promoting the degradation of STING	^142^
STING	Cylindromatosis	Deubiquitination	K150; Removal of ubiquitylation of RNF5	^143^
STING	RNF26	Ubiquitylation	K150; Protecting STING from degradation competing with RNF5	^144^
STING	USP18/USP20	Deubiquitination	Preventing degradation of STING	^145^
STING	OTUD5	Deubiquitination	Promoting stability of STING	^146^
STING	TRIM56	Ubiquitylation	K150; Promoting STING dimerization and interaction with TBK1	^147^
STING	TRIM32	Ubiquitylation	K20, K224, and K236; Promoting STING dimerization and interaction with TBK1	^148^
STING	Mitochondrial E3 ubiquitin protein ligase 1	Ubiquitylation	K224; Promoting the translocation of STING from ER to Golgi	^149^
STING	RNF115	Ubiquitylation	K20, K224, and K289; Promoting the translocation of STING from ER to Golgi	^150^
STING	MYSM1	Deubiquitination	K150; Preventing excessive activation of STING	^151^
STING	INSIGI/AMFR	Ubiquitylation	K137, K150, K224, and K236; Constructing an anchoring platform to recruit TBK1 to promote TBK1 activation	^152^
STING	USP13	Deubiquitination	Inhibiting the recruitment of TBK1 to STING	^153^
STING	USP21	Deubiquitination	Block the phosphorylation of IRF3 and its transport to the nucleus	^154^
STING	USP35	Deubiquitination	Inhibiting the activity of STING	^155^
STING	TBK1	Phosphorylation	Ser366; Promoting recruitment and phosphorylates IRF3	^107^, ^156^
STING	TRIM38	Sumoylation	K338; Inhibiting the degradation of STING through chaperone‐mediated autophagy (CMA) pathway	^129^
STING	ULK1/2	Phosphorylation	S365; Reducing the activity of STING	^122^
STING	SENP2	Desumoylation	K337; Promoting the degradation of STING through the CMA pathway	^129^
STING	Palmitoyl transferase	Palmitoylation	Cys88, Cys91; Promoting the assembly of multimeric complexes and the recruitment of downstream signaling factors	^99^, ^100^
STING	4‐HNE	Carbonylation	Preventing the normal palmitoylation of STING activation	^157^
STING	NO_2_‐FAs	Nitroalkylation	Preventing the normal palmitoylation of STING activation	^158^

Abbreviations: 4‐HNE, 4‐hydroxynonenylaldehyde; Akt, protein kinase B; CCP, cytoplasmic carboxypeptidase; INSIGI/AMFR, ER membrane protein insulin‐induced gene 1/autocrine motion factor receptor; IRF3, IFN regulatory factor 3; MYSM1, Myb‐like, SWIRM, and MPN domains 1; NO_2_‐FAs, nitro‐fatty acids.; OTUD5, Ovarian tumor domain deubiquitinating enzyme 5; PTM, posttranslational modification; RNF, ring finger; SENP, Sentrin/SUMO‐specific protease; TBK1, TANK‐binding kinase 1; TRIM, tripartite motif; TTLL, tubulin tyrosine ligase‐like; ULK1/2, Unc‐51‐like kinase 1/2; USP, ubiquitin‐specific protease.

### 5.1 PTM of cGAS

Accurate recognition of immune stimulatory DNAs through cGAS is essential for activating the innate immune response correctly. Therefore, it is necessary to strictly regulate cGAS activity to maintain immune homeostasis in physiological and pathological processes. cGAS is subject to different types of PTMs, including phosphorylation, ubiquitination, sumoylation, glutamylation, proteolysis, acetylation, and methylation, which play an important role in the stability, enzyme activity, and ligand binding ability of cGAS (Table 1).

Phosphorylation and dephosphorylation of cGAS are essential for regulating the subcellular activity and/or localization of cGAS. B‐lymphoid tyrosine kinase causes cGAS to be phosphorylated at Y215 to maintain cytoplasmic localization in resting cells. In contrast, immunostimulatory DNA induces dephosphorylation and subsequent nuclear translocation of cGAS.^116^ Protein kinase B (Akt) has shown an inhibitory effect on cGAS‐mediated antiviral immunity, and it phosphorylates cGAS at S305 (S291 of mouse cGAS) to produce a negatively charged phosphate group, which blocks the binding and catalysis of cGAS for ATP and GTP, leading to inhibition of enzyme activity and reduction of cGAMP and IFN‐β production.^117^ Thus, cGAS phosphorylation at this site leads to elevated HSV‐1 titers after infection. In the resting state, protein phosphatase 6 dephosphorylates cGAS at S435 (human) or S420 (mouse) but dissociates from cGAS during DNA virus infection, resulting in phosphorylation of cGAS and enhancing the binding affinity and enzyme activity of cGAS‐GTP.^118^ The cyclin‐dependent kinase 1 (CDK1)‐cyclin B kinase complex phosphorylates the S305 site of cGAS, thereby eliminating cGAMP synthesis in mitotic cells, and this phosphorylation is reversed by protein phosphatase 1 (PP1) during cell division.^119^ Moreover, cGAS is hyperphosphorylated by mitotic Aurora kinase B at S13, S37, S64, T69, T91, S116, S129, and S143 of the N‐terminus, which is dephosphorylated by PP1 or protein phosphatase 2A (PP2A) at the end of mitosis. In addition, hyperphosphorylation of the cGAS N‐terminus blocks its recognition of chromatin DNA, thereby inhibiting its activity in mitosis.^120^

Protein ubiquitination is an essential PTM. cGAS ubiquitination is reversely modified by several E3 ligases or deubiquitinating enzymes,^121^, ^122^ which play a key role in regulating its stability and/or enzyme activity. The E3 ligase TRIM56 containing the tripartite motif (TRIM) promotes monoubiquitination of cGAS at K335 to enhance its dimerization, which is of great significance for DNA binding activity, the production of cGAMP and IFN‐I, and anti‐DNA virus immunity.^123^ The E3 ligase RNF185 containing the ring finger (RNF) protein promotes K27 polyubiquitination at position K137/384 of cGAS, thus promoting its enzyme activity, which enhances the production of IFN during HSV‐1 virus infection.^124^ E3 ligase‐mediated K48‐linked ubiquitination of cGAS leads to its degradation through the autophagy pathway, but the mechanism still needs to be clarified. After DNA virus infection, IFN‐induced TRIM14 recruits ubiquitin‐specific protease 14 (USP14) to cut the K48‐linked polyubiquitin chain of cGAS, thus inhibiting the p62‐dependent degradation of cGAS.^115^, ^125^ The deubiquitinating enzymes USP27X and USP29 interact directly with cGAS and remove the K48‐linked polyubiquitination chain from cGAS, thus promoting the stability of cGAS.^126^, ^127^

Sumoylation is another PTM to regulate the stability and enzyme activity of cGAS. A small ubiquitin‐like modifier (SUMO) is coupled to lysine residues 335, 372, and 382 of cGAS, inhibiting its binding to DNA, oligomerization, and nucleotidyltransferase activity.^128^ In contrast, the Sentrin/SUMO‐specific protease SENP7 catalyzes the desumoylation of cGAS and enhances its activation to reverse this inhibition. In uninfected cells or at the early stage of virus infection, TRIM38 targets cGAS to carry out sumoylation at K464 and K217 to prevent K48‐linked polyubiquitination and subsequent proteasomal degradation of cGAS. In the late stage of infection, SENP2 catalyzes the desumoylation of cGAS, making it polyubiquitinated and then degraded by the proteasome.^129^

Protein glutamylation is an ATP‐dependent PTM catalyzed by glutamylase and removed by carboxypeptidase. The tubulin tyrosine ligase‐like (TTLL) enzyme protein TTLL6 catalyzes the glutamylation of cGAS at E272, which hinders its DNA binding ability, while TTLL4 induces the single glutamylation of cGAS (E302) to block its enzyme activity,^115^, ^130^ which reduces the synthesis of cGAMP and hinders the induction of DNA‐stimulated IFN in HSV‐1 infection. In contrast, cytoplasmic carboxypeptidase CCP6 and CCP5 can remove polyglutamylation and monoglutamylation, restoring the DNA binding and catalytic activity of cGAS,^130^ and promoting the induction of transcription factors IRF3 and IFN. In addition, the lack of CCP5 or CCP6 leads to increased susceptibility to DNA viruses.^4^

Microbial inflammation is mediated by the activation of inflammatory Caspases (Caspase‐1 and Caspase‐4/5 in humans or Caspase‐11 in mice).^131^ For example, the inflammatory cell form in sepsis is mediated by the activation of Caspase 1 induced by inflammatory corpuscles. Activated Caspase 1 binds directly and cuts cGAS at D140/D157 to prevent cGAS from being activated by DNA stimulation.^132^ The proapoptotic BCL‐2 homologous antagonist and BCL‐2‐related X protein (BAX) trigger the release of mtDNA into the cytoplasm to be recognized by and to activate cGAS.^133^, ^134^ However, Caspase 3 activated in apoptotic cells cleaves cGAS at D319 to inhibit its activation.^135^ Obviously, the above proteolysis inhibits the activation of the cGAS–STING signal.

In addition, acetylation and methylation are also important PTM regulators of cGAS. Aspirin acetylates cGAS at K384, K394, and K414, inhibiting DNA‐induced cGAS activation.^136^ In contrast, the acetylation of lysine acetyltransferase 5 at the N‐terminal domains K47, K56, K62, and K83 of cGAS can promote its DNA binding ability, thus enhancing innate immunity to DNA viruses.^137^ Protein arginine methyltransferase 5 catalyzes the methylation of cGAS at R124, which blocks the DNA binding ability of cGAS to attenuate the antiviral immune response.^138^

### 5.2 PTM of STING

The activation degree and duration of IFN‐I and proinflammatory cytokines are crucial for the host to resist pathogenic microbial infection and maintain autoimmunity stability. Therefore, the production of these molecules must be strictly regulated to prevent their overactivated immune response.^139^ STING is a key molecule that controls the production of DNA‐induced IFN‐I and proinflammatory cytokines. Thus, the regulatory mechanism of STING in the host is tight and complex. Like cGAS, STING's stability, localization, and activation are also regulated by PTMs, including ubiquitination, sumoylation, phosphorylation, and palmitoylation (Table 1).

The E3 ubiquitin ligases RNF5 (K150),^140^ TRIM29 (K288 and K337),^141^ and TRIM30 α (K275)^142^ mediate the K48‐linked polyubiquitination of STING, promoting its degradation, and inhibiting the innate immune response to DNA viruses. In contrast, the ubiquitination enzyme cylindromatosis (CYLD) removes the RNF5‐mediated K48‐linked polyubiquitin chain from STING in the Golgi apparatus and enhances the antiviral innate immune response.^143^ More interestingly, RNF26, another E3 ubiquitin ligase, competes with RNF5 by catalyzing cGAS K11‐linked polyubiquitination at K150, thereby protecting STING from degradation and maintaining rapid and effective induction of IFN‐I and proinflammatory cytokines after virus infection.^144^ USP18 recruits USP20 to uncouple the K48‐linked polyubiquitin chain from STING, preventing STING from proteasomal degradation, and promoting STING's stability and production of IFN‐I and proinflammatory cytokines during DNA virus infection.^145^ Ovarian tumor domain deubiquitinating enzyme (OTUD) 5 interacts with STING to cleave its K48‐linked polyubiquitin chain, promoting its stability,^146^ which is essential for STING‐mediated antiviral and antitumor immunity.

TRIM56 (K150) and TRIM32 (K20, K224, and K236) mediate the K63‐linked ubiquitination of STING to assist in STING dimerization and interact with TBK1.^147^, ^148^ Similarly, mitochondrial E3 ubiquitin protein ligase 1 (K224) and RNF115 (K20, K224, and K289) combine and catalyze the K63‐linked polyubiquitination of STING to promote its activation and translocation from ER to the Golgi apparatus.^149^, ^150^ In contrast, cytoplasmic DNAs stimulate the expression of Myb‐like, SWIRM, and MPN domains 1 (MYSM1) protein to interact with STING, cutting the K63‐linked ubiquitination chain of STING at K150 to limit the overactivation‐of‐STING.^151^ In addition, ER membrane protein insulin‐induced gene 1 (INSIGI) bridges the autocrine motion factor receptor (AMFR) and STING, which promote the K27‐linked multiubiquitination of STING catalyzed by AMFR at K137, K150, K224, and K236 and forms an anchor platform for recruiting TBK1, thus promoting the activation of TBK1 to enhance the immune response.^152^ In HSV‐1 infection, ubiquitin‐specific protease 13 (USP13) uncouples the K27‐linked polyubiquitin chain from STING to inhibit the recruitment of TBK1 to STING, thus negatively regulating the antiviral response of cells.^153^ Porcine circovirus 2 (PCV2) infection activates the phosphorylation of USP21 mediated by the p38‐MAPK signaling pathway to inhibit the K63‐ubiquitination of STING, thus preventing the phosphorylation of IRF3 and its transport to the nucleus, leading to an increased risk of PCV2 infection.^154^ In cancer cells, activated STING promotes its binding with USP35 in a manner dependent on the phosphorylation of STING. USP35 can directly remove K6‐, K11‐, K27‐, K29‐, or K63‐linked ubiquitination and inactivate STING.^155^ Therefore, the upregulation of USP35 may be the restriction mechanism of STING activity in cancer cells.

In addition to ubiquitination, sumoylation and phosphorylation are also important for the stability and activation of STING. In the early stages of STING activation, STING forms oligomers to recruit and activate TBK1 (autophosphorylation). Then, TBK1 phosphorylates STING at Ser366 as the docking site for interaction with IRF3 and subsequent phosphorylation.^107^, ^156^ TRIM38 mediates the sumoylation of STING at K338 to inhibit the degradation of STING through chaperone‐mediated autophagy (CMA). TBK1 is delivered to the endosome/lysosome compartment in the late stage, and the transcription factors IRF3 and NF‐κB are activated. Then, cGAMP activates ULK1/2 kinase, resulting in the downregulation of STING phosphorylation and activity at S365.^122^ Phosphorylated STING is further translocated to the perinuclear microsomes, which recruit SENP2 to desumoylate STING at K337 and promote its degradation through the CMA pathway. In contrast, TBK1 is a stronger kinase of STING than ULK1, and TBK1 phosphorylates IRF3 only at S396, which is a sign of IRF3 activation.^129^

In addition, activated STING translocates to the Golgi apparatus and is palmitoylated at Cys88 and Cys91 by palmitoyl transferases rich in Asp‐His‐His‐Cys (DHHC), which is important for subsequent STING polymer assembly and recruitment of downstream signal molecules.^99^, ^100^ Interestingly, during viral infection, lipid peroxidation causes the accumulation of 4‐hydroxynonenylaldehyde (4‐HNE) and nitro‐fatty acids (NO_2_‐FAs), resulting in carbonylation and nitro‐alkylation of STING, thus preventing its activation of the normal palmitoylation process.^157^, ^158^

In summary, although the PTMs to regulate cGAS/STING signaling pathway remains fully elucidated, understanding these regulating mechanisms may provide potential targets for drug development to treat cGAS/STING‐related diseases (Table 1).

## 6 ACTIVATION AND INHIBITION OF cGAS–STING UNDER THE PATHOLOGICAL CONDITIONS

Regulation of cGAS–STING signaling pathway occurs not only during the normal physiological state, but also under a pathological state. Under pathological conditions, the cGAS–STING signal may be inhibited or over‐activated, causing sustained damage to the host body. Here, we summarize the activation and inhibition mechanisms of cGAS–STING signaling pathway, and we discuss its influence on the occurrence and progression of disease under various pathological conditions.

### 6.1 cGAS–STING signaling pathway in viral infection

**FIGURE 4:**
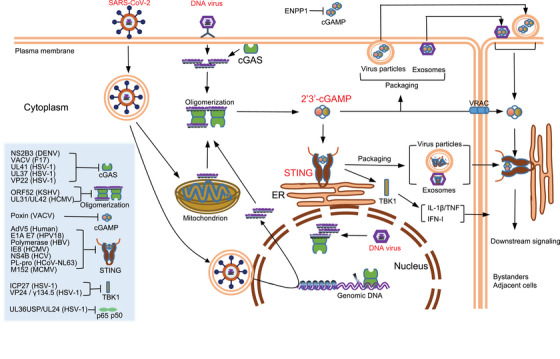
Activation and inhibition of the cGAS–STING signaling pathway in viral infection. The DNA virus (red font) is sensed by cGAS localized to the plasma membrane when it releases its DNA into the cytoplasm, and by cGAS localized to the nucleus when it is released into the nucleus. SARS‐CoV‐2 (red font) activates the cGAS–STING signal by triggering chromatin DNA damage and mtDNA release into the cytoplasm. In viral infection, 2′,3′‐cGAMP and STING (red font) are key molecules. In addition to activating STING, 2′,3′‐cGAMP can enter into bystanders or adjacent cells via VRAC or by be encapsulated into viral particles or exosomes to rapidly activate STING‐dependent antiviral immune response. Activation of STING signaling pathway leads to the production of IFN‐I and proinflammatory cytokines, which can further induce a broad antiviral immune response. In addition, activated STING can also be encapsulated into viral particles or exosomes to enter into bystanders or adjacent cells. Notably, a variety of viruses can use secreted proteins to inhibit multiple molecules or processes of the cGAS–STING signaling pathway (bottom left, light blue), such as cGAS and oligomerization, cGAMP, STING, TBK1, and NF‐κB (p65/p50), thus suppressing antiviral immune response. SARS‐CoV‐2, severe acute respiratory syndrome coronavirus 2; VRAC, volume‐regulated anion channel.

When a pathogenic microorganism infects the host, exposure to exogenous DNAs in the host cytoplasm can successfully and effectively activate cGAS–STING signaling pathway, and the consequent production of large amounts of IFN‐I is conducive to eliminating the pathogenic microorganism. However, the strong selective pressure exerted by the cGAS–STING signaling pathway on pathogens (especially viruses) has prompted some pathogens to infect the host using successful strategies that counter or evade the monitoring of the cGAS–STING signaling pathway (Figure 4).^159^

#### 6.1.1 Activation of the cGAS–STING signaling pathway to initiate host defense

##### Host cells sense the viral infection

The distribution of cGAS in the host cell covers almost all of the sites where a pathogen would release its DNA, optimizing the monitoring sensitivity of the cGAS–STING signaling pathway and facilitating prompt initiation of innate immunity to the pathogen. When infected with a virus, the cGAS on the plasma membrane captures enough agonist (virus DNA), and more is released from the plasma membrane to transmit signals in the cytoplasm.^160^, ^161^ The cGAS located in the nucleus only recognizes virus that exposes its DNA in the nucleus. Importantly, only when the concentration of DNA reaches a certain threshold is activation of cGAS triggered, leading to downstream activation of the cGAS–STING signaling pathway.^162^ Viral infection eventually destroys homeostasis in the host cell, and exposure to increasing DNA concentrations activates the cGAS–STING signaling pathway quickly. For example, infection with HSV‐1 and dengue virus (DENV) result in leakage of mtDNA into the host cell cytoplasm, rapidly activating the cGAS–STING‐dependent antiviral innate immune response.^70^, ^163^ Moreover, in the case of infection, genomic instability may also lead to an increased risk of DNA exposure and enhanced innate immunity against pathogens.^78^, ^164^ Other changes in the intracellular environment during virus infection may facilitate the detection of viral DNA, including cytoplasmic acidification and an increase in the concentration of cytosolic Mn^2+^ following mitochondrial membrane potential damage, both of which increase the sensitivity of cGAS to dsDNA.^165^ In summary, targeted distribution of the DNA recognition molecule cGAS within the cell and the ability of the host cell to integrate multiple signals greatly contribute to improving the monitoring sensitivity of the cGAS–STING signaling pathway.

##### Signal transmission

After recognizing viral DNA, cGAS catalyzes the formation of cGAMP, which binds and activates STING, ultimately leading to the expression of IFN‐I and proinflammatory cytokines. It is worth noting that host cells have adopted counter‐strategies conducive to antiviral signal transmission at multiple stages of this signaling pathway.^7^ First, cGAMP can be integrated into virus particles and transferred to newly infected cells to induce a STING‐dependent antiviral response,^166^, ^167^ which means that cGAMP can start innate immune signal transduction faster and more effectively without the activation of cGAS. In addition, cGAMP can be transferred between cells during virus infection by other mechanisms. When infected by HSV‐1, 2′,3′‐cGAMP is transported into bystander cells via the volume‐regulated anion channel (VRAC). Notably, inflammatory cytokines related to virus infection (such as IL‐1β and TNF) enhance the activation of VRAC, thus facilitating cGAMP‐dependent transmission of the immune response to bystander cells.^168^ Moreover, STING can also be transferred to adjacent cells. For example, STING has been identified in exosomes and virus particles after HSV‐1 infection, and these can be transferred to adjacent cells to activate the immune response.^169^ In a similar manner, STING packaged into virus particles can limit the transmission of herpes virus between host cells. In addition, the effector molecules produced by the cGAS–STING signaling pathway, such as IFN‐I and IL‐1β, also transmit and expand the antiviral immune response. Studies have shown that IL‐1β signal transduction causes mitochondrial stress and mtDNA release, activating the cGAS–STING signal and subsequently promoting immune protection in bystander cells.^29^, ^109^

##### Activation of the cGAS–STING signaling pathway by RNA virus

The cGAS–STING signaling pathway plays a significant role in the silencing of viral DNA. Interestingly, it also plays an important role in host innate immunity against certain single‐stranded RNA viruses. For example, cGAS knockout mice are more likely to be infected with sense West Nile virus (a single‐stranded RNA virus).^29^ Furthermore, STING‐deficient mice also demonstrate greater susceptibility to RNA virus. Indeed, STING‐deficient cells do not produce a strong innate immune response to RNA viruses such as vesicular stomatitis virus and SeV.^102^ Numerous studies have also shown that the cGAS–STING signaling pathway is activated during RNA virus infection. Negative‐stranded RNA paramyxovirus, Nipavirus (NiV), and measles virus (MeV) all trigger a cGAS–STING signal. In human and mouse cells, deletion of cGAS or STING reduced IFN‐I production and enhanced Paramyxovirus infection. Phosphorylation and ubiquitination of STING were also observed during viral infection, confirming that NiV and MeV activated the cGAS–STING signaling pathway.^170^

It is worth noting that the severe acute respiratory syndrome coronavirus 2 (SARS‐CoV‐2), responsible for the COVID‐19 pandemic also activates the innate immune response through the cGAS–STING signaling pathway. During SARS‐CoV‐2 infection, cell fusion caused by the binding of SARS‐CoV‐2 spike protein to host cell angiotensin converting enzyme 2 triggers the shuttling of chromatin DNA to the cytoplasm, where it is recognized by cGAS. As expected, this catalyzes the production of 2′,3′‐cGAMP, which is associated with STING activation, and effectively inhibits viral replication.^171^, ^172^ SARS‐CoV can also indirectly stimulate the T‐cell response through the cGAS–STING signaling pathway. When SARS‐CoV‐2 virus induces DNA damage in cells adjacent to T cells, the cGAS–STING signaling pathway is activated in CD4^+^ and CD8^+^ T cells after the cGAMP produced is transferred to the adjacent T cells.^173^ Recent studies have found that SARS‐CoV‐2 infection also activates cGAS–STING signal transduction in macrophages and endothelial cells by releasing mtDNA, resulting in cell death and IFN‐I production. However, although the rapid induction of IFN‐I limits the spread of the virus, a continuous increase in IFN‐I levels during the late stage of infection is related to abnormal inflammation and adverse clinical outcomes.^174^, ^175^, ^176^ In conclusion, cGAS and STING may limit further RNA virus infection by a mechanism different from resisting DNA virus infection.

#### 6.1.2 Pathogen evasion from the cGAS–STING signaling pathway

The cGAS–STING signaling pathway monitors cytoplasmic DNA and triggers a strong immune response, thus limiting the replication of multiple pathogens. However, virus genetic material is relatively simple and prone to mutations beneficial to its survival. Therefore, under the strong selection pressure from host cells, some viruses (e.g., HSV‐1^177^) have successfully established escape strategies for the cGAS–STING signaling pathway.

##### Prevention or downregulation of cGAS activation

Because cGAS is the key DNA recognition sensor, so many viruses have evolved escape mechanisms for cGAS. First, some viruses can block activation of the cGAS–STING signaling pathway by blocking the recognition of viral DNA by cGAS in host cells.^160^, ^178^ For example, when the herpes virus infects target cells, it shields the DNA in a viral capsid before reaching the nucleus, and it has increased capsid stability through mutation to prevent the DNA from being exposed to the cytoplasm (thereby avoiding activation) of cGAS.^179^, ^180^ Second, some viruses induce cGAS cleavage or degradation. For example, the DENV protease complex NS2B3 can cleave cGAS to prevent it from being activated by mtDNA during infection,^181^ while the Vaccinia virus (VACV) F17 protein causes an imbalance in the mTOR signaling pathway, leading to enhanced degradation of cGAS (and impaired cytosolic DNA‐dependent activation of cGAS–STING signaling at later stages of infection).^182^ HSV‐1 UL41 selectively degrades cGAS mRNA through its RNase activity, resulting in a decrease in the cGAS protein levels (and a decrease in the ability of host cell to sense viral DNA).^183^ Another strategy involves the virus particles carrying immune antagonists that block activation of cGAS. KSHV ORF52 (also known as KicGAS) effectively inhibits cGAS–DNA phase separation to block the activation of cGAS in vitro and in cells.^184^ Human cytomegalovirus (HCMV) expresses UL31 and UL42 to interfere with cGAS‐DNA binding and higher‐order complex formation.^185^, ^186^ In addition, HSV‐1 inner tegument protein VP22 interacts with cGAS and inhibits its enzymatic activity,^187^ while UL37 has been shown to deamidate the essential Asn residues in human and mouse cGAS, resulting in impaired cGAS activity.^188^

##### Degradation of 2′,3′‐cGAMP

The second messenger, 2′,3′‐cGAMP, can be transmitted through intercellular junctions to activate STING signal transduction in adjacent uninfected bystander cells.^189^ It can also be packaged into newborn virus particles to drive a rapid immune response when new target cells are infected.^166^, ^167^ Therefore, given the important biological function of 2′,3′‐cGAMP, it is also the target of virus attack. For example, VACV and other related vaccinia viruses can encode a vaccinia virus nuclease, Poxin, which degrades 2′,3′‐cGAMP to block the activation of the cGAS–STING signaling pathway.^190^ In mammalian tissues and plasma, extracellular nucleotide‐pyrophosphatase/phosphodiesterase family member 1 has been demonstrated to be the main component that degrades 2′,3′‐cGAMP.^191^ It can be concluded that the degradation of 2′,3′‐cGAMP is a more extensive mechanism for controlling the transmission of innate immune signals.

##### Prevention of STING activation

STING is a key protein in the cGAS–STING signaling pathway, so the viruses have also evolved a series of escape strategies against STING, including binding and blocking the activation of STING, interfering with the PTM of STING, degrading STING to block the assembly of STING and interfering with the transport of STING.^192^ Human AdV5 E1A and papillomavirus 18 (HPV18) E7 bind STING through their Leu‐X‐Cys‐X‐Glu (LXCXE) motif and block downstream signal transduction.^193^ HBV virus polymerase prevents the K63‐polyubiquitination of STING, ultimately blocking the production of IFN‐β and antiviral immunity in hepatocytes.^194^ The HCMV IE86 protein promotes STING degradation in the proteasome, thus limiting the activation of downstream signal transduction.^195^ Two RNA viral factors, hepatitis C virus (HCV) NS4B and coronavirus papain‐like protease PL‐pro, interfere with STING oligomerization.^196^, ^197^ In addition, M152 from mouse cytomegalovirus delays the transport of STING from the ER to the Golgi apparatus, thus interfering with IRF3 signal transduction and the antiviral IFN‐I response.^192^

##### Suppressing downstream signal transduction

As the main effectors of the cGAS–STING signaling pathway, and the key regulatory factors of innate immunity, IRF3 and NF‐κB are strongly inhibited by viral proteins. For example, HSV‐1 ICP27 interacts with STING in a manner that depends on the activity of TBK1, resulting in decreased phosphorylation of IRF3 and impaired activation.^198^ VP24 and γ 134.5 destabilize the interaction between TBK1 and IRF3, ultimately inhibiting IRF3‐mediated transcription.^199^, ^200^ The HSV‐1 deubiquitinating enzyme UL36USP stabilizes IκBα, an inhibitor of NF‐κB, while UL24 inhibits translocation of the NF‐κB subunit to the nucleus. Hence, these two proteins inhibit the activity of NF‐κB.^201^, ^202^ Finally, β‐catenin protein is an important factor involved in enhancing the transcription of IFN‐I in the cGAS/STING signaling pathway, is antagonized by HSV‐1 US3 protein via its kinase activity.^203^

### 6.2 cGAS–STING signaling pathway in cancer

**FIGURE 5:**
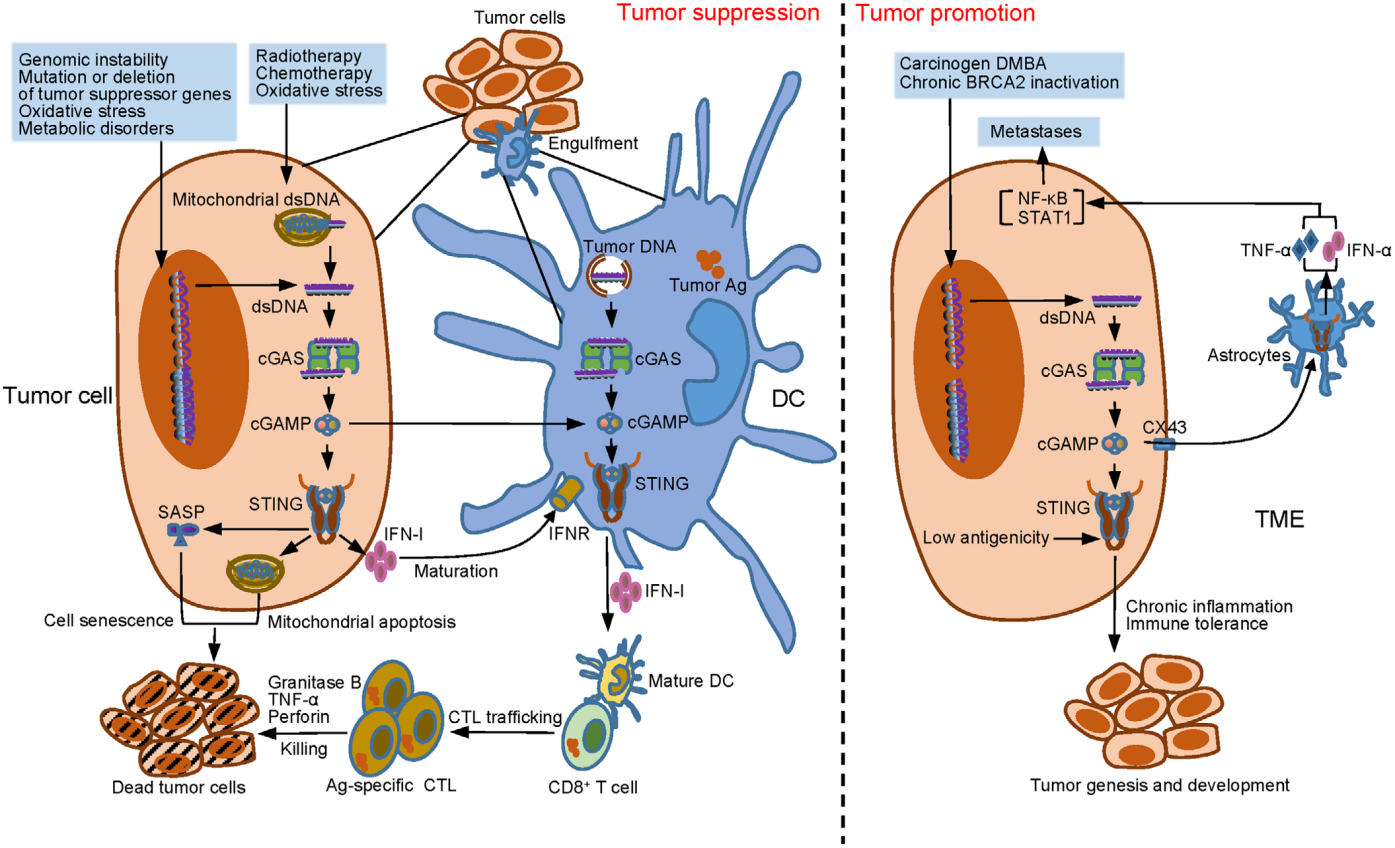
Tumor suppression and promotion of the cGAS–STING signaling pathway. Tumor suppression: in tumor cells, the genomic DNA exposure caused by genomic instability, mutation or deletion of tumor suppressor genes, oxidative stress, and metabolic disorders, and the mitochondrial DNA leakage caused by radiotherapy, chemotherapy, and oxidative stress, can all activate the cGAS–STING signal in the cytoplasm. In addition, cGAS–STING signal can promote mitochondria‐mediated apoptosis and SASP‐induced cell senescence. More importantly, IFN‐I promotes the maturation of DCs by binding to receptors. Then, mature DCs that have engulfed tumor antigens contact CD8^+^ T cells and promote the generation of tumor antigen‐specific CTLs to eliminate tumor cells directly. In addition, the phagocytosis of tumor cell DNA by DCs and the transfer of cGAMP from tumor cells to DCs can both activate STING‐dependent antitumor immune response. Tumor promotion: carcinogen DMBA and chronic BRCA2 inactivation lead to genomic DNA fragmentation, triggering the activation of the cGAS–STING signal, which induces a chronic inflammatory response and eventually leads to tumorigenesis. Moreover, the low antigenicity of tumor cells induce tolerogenic immune response via the STING signaling pathway and promote tumor development. In addition, cGAMP enters astrocytes through the gap junction formed by CX43 to activate STING signaling pathway and induce the production of TNF‐α and IFN‐α, activating the NF‐κB and STAT1 signaling pathways in tumor cells to promote tumor cell metastasis. BRCA2, breast cancer susceptibility gene 2; CTL, cytotoxic T lymphocytes; CX43, connexin 43; DC, dendritic cell; DMBA, 7,12‐dimethylbenzene (a) anthracene; SASP, senescence‐associated secretory phenotype.

The cGAS–STING signaling pathway has a pivotal function in immune activation, and numerous reports provide evidence that the cGAS–STING pathway plays a vital role in inhibiting tumor progression, for which many immunotherapies have been developed. However, a growing number of findings suggest a diversity of its roles in tumorigenesis, including functions that promote tumorigenesis and progression (Figure 5).^204^, ^205^, ^206^, ^207^, ^208^, ^209^

#### 6.2.1 Immune surveillance and antitumor immunity

Due to their continuous clearance from the immune system, cancer cells acquire abnormal characteristics, such as uncontrolled proliferation, through the accumulation of mutations. However, during the clearing process, the immune system recognizes cancer cells as “foreign cells” and subsequently targets them. Thus, there is an immune monitoring system for cancer.^210^ Under normal circumstances, eukaryotic DNA is not exposed in the cytoplasm to avoid causing autoimmunity.^211^ However, DNA leaks into the cytoplasm abnormally in tumor cells,^212^, ^213^ activating the cGAS–STING signaling pathway, ultimately inducing IFN‐I in DCs and endothelial cells to initiate antitumor immunity. Thus, cGAS is extremely important for the recognition of tumor cell DNA. However, activation of the cGAS–STING signaling pathway is dependent on the exposure of tumor cell DNA within the cytoplasm, and this process requires some consideration. Studies have found that cancer cells often exhibit genomic instability, the mutation or deletion of tumor suppressor genes, oxidative stress, and metabolic disorders.^214^ As a result of these strong stimuli, nuclear and mitochondrial DNA easily leak into the cytoplasm in the form of micronuclei, chromatin fragments, and/or free telomere DNA. cGAS molecules in these tumor cells then recognize the DNA and generate cGAMP (subsequently activating STING).^74^, ^78^, ^215^ It should be noted that the cGAMP generated can transfer to adjacent nontumor cells to activate their STING‐dependent immune response.^210^, ^216^ In addition, DNA exposure caused by exogenous stimulation such as chemotherapy and radiotherapy, mtDNA leakage induced by oxidative stress, and mitochondrial membrane permeability caused by anticancer therapy, help to increase the concentration of abnormal cell DNA, facilitating the rapid activation of cGAS.^67^, ^217^ Finally, DNA derived from apoptotic cells, exosomal DNA, and transposable elements have also been shown to induce the activation of cGAS–STING signaling pathway in tumor cells.^218^, ^219^

IFN‐I plays a key role in activating antitumor immunity.^204^ IFN‐I stimulates the interaction between antigen‐presenting cells (APCs) and tumor cells, and the antigen (Ag) produced by these tumor cells is engulfed by APCs (such as DCs). APC uptake of tumor DNA activates the cGAS–STING signal and induces the production of IFN‐I and cytokines (such as IL‐12), as well as the expression of costimulatory molecules (such as CD40, CD80, and CD86). These APCs subsequently present the tumor cell derived Ag to cytotoxic CD8^+^ T lymphocytes (CTCL). CTCL recognition of this Ag induces its activation and leads to the secretion of cytokines, including tumor necrosis factor α (TNF‐α), perforin, and granzyme B, which work together to kill target tumor cells. In addition to the direct effects, the cGAS–STING signaling pathway also plays an important role in inducing cancer cell senescence and apoptosis. It has been found that cell senescence is an important tumor inhibition mechanism, and cytosolic DNA activation of the intrinsic cGAS–STING signaling pathway is believed to play a role in cell senescence.^220^ The senescence‐related secretion phenotype (SASP) is the general term for various proteins secreted by senescent cells, such as cytokines, chemokines, and growth factors. cGAS is the key molecule linking DNA damage, SASP, and senescence.^221^ In aging cells, an accumulation of DNA in the cytoplasm activates the cGAS–STING signaling pathway, producing SASP and ROS and promoting sustained cell aging.^222^ SASP can inhibit abnormal cell growth, prevent tumor formation during cell aging, and recruit immune cells to stimulate local immune responses to eliminate senescent cells and cells expressing oncogenes.^223^ The cGAS–STING signaling pathway also promotes cell apoptosis through of apoptosis‐regulating factor and IRF3. After activation of the STING signaling pathway, expression level of the antiapoptotic protein BCL2 is downregulated, while expression level of the proapoptotic protein BCL2‐related X (BAX) is upregulated, this change in the levels of these two proteins mediates a permeability change in the mitochondrial outer membrane (driven by Caspase‐9), and the activation of Caspase‐3 to promote cell apoptosis.^224^ In addition, high level of STING expression in T cells enhances the proapoptotic effect of two BH3 proteins, Noxa and Puma. The proapoptotic response from STING also plays an important role in culling malignant or abnormal T cells,^225^ a potential therapeutic method for T‐cell‐derived malignant tumors. Consistent with this view, patients with STING‐associated vasculopathy with onset in infancy (SAVI) carry an overactive STING mutant, and T‐cell apoptosis in their body is significantly increased.^226^ In conclusion, studying the antitumor mechanism of the cGAS–STING signaling pathway provides new ideas and directions for treating cancer.

#### 6.2.2 Protumor effect

In conclusion, activating of the cGAS–STING signaling pathway upregulates IFN‐I production and exerts antitumor effects by mediating innate immune responses. However, chronic stimulation of cGAS–STING signaling pathway may lead to abnormal inflammatory responses and even inflammation‐related cancers. For example, 7,12‐dimethylbenzene (a) anthracene (DMBA) is a carcinogen that activates the cGAS–STING signal and promotes skin tumorigenesis in mice by inducing DNA breaks.^220^, ^227^ Moreover, inactivation of the breast cancer susceptibility gene 2 (BRCA2) leads to impaired DNA repair and micronucleus accumulation, which activates the cGAS–STING signaling pathway, leading to cell cycle arrest, increased IFN signaling and cell death. However, during chronic inactivation of BRCA2, and therefore chronic cGAS stimulation, not only is ISG upregulated, but cell cycle progression is restored, thereby promoting the survival of mutant cells.^86^, ^228^, ^229^ In addition, cGAS–STING signal promotes cancer cell metastasis through intercellular paracrine signal transduction in the tumor microenvironment (TME).^230^ Protocadherin 7, expressed by breast and lung cancer cells, promotes the polymerization of cancer cell–astrocyte gap junctions consisting of connexin 43 (CX43), enabling the transfer of cGAMP from cancer cells to astrocytes. Subsequent STING activation in astrocytes leads to the secretion of TNF‐α and IFN‐α, which activate NF‐κB and STAT1 signaling in cancer cells, ultimately promoting the survival and growth of metastatic cancer cells in the brain.^231^ It has also been found that cell membrane fusion mediates STING activation, and the surge in membrane fusion during the shedding of exosomes from cancer cells may also lead to STING activation to promote cancer cell metastasis.^232^ In conclusion, dissecting the role of the cGAS–STING signaling pathway in antitumor immunity and tumorigenesis should provide important insights into the search for cancer therapeutic targets.

#### 6.2.3 Expression levels of cGAS and STING in tumors

Due to the continuous leakage of tumor cell DNA into the cytoplasm, the expression levels of genes in the cGAS–STING signaling pathway of tumor cells may be regulated, thus avoiding an antitumor immune response. For example, after the deletion of the 9p chromosome containing the IFN gene cluster, the tumor can undergo NF‐κB signal downstream of STING without triggering the IFN response. In addition, the immune escape mechanism may involve the downregulation of STING expression level, and this is common in gastric cancer, colorectal cancer, and advanced cancers.^216^, ^233^ In comparison with its expression in normal gastric epithelium, STING expression level in gastric cancer specimens was significantly decreased. In gastric cancer cell lines, dsDNA stimulation or cGAMP can reduce the expression level of STING, and STING knockdown can increase the growth, migration, and invasion of tumor cells.^234^ This suggests that low STING expression may reduce the antitumor activity of the cGAS–STING signaling pathway. In low antigenic tumors such as Lewis lung cancer, sensing of cytoplasmic DNA and activation of STING in normal tissue promote tumor growth rather than regression, suggesting that the downstream effects of STING signaling pathway activation are highly dependent on the antigenicity of tumor cells.^234^ Thus, activation of the STING signaling pathway may induce a tolerant immune response and promote tumor progression under conditions of decreased antigenicity. STING inhibition provides another way for cancer cells to escape activation of the cGAS–STING signaling pathway. In cancer cells with HER2 mutations, HER2 combines with STING and recruits the kinase AKT1 to phosphorylate TBK1, thus preventing the interaction between STING‐TBK1 and TBK1‐IRF3, ultimately shielding cancer cells from host antitumor immunity.^235^ While some data suggest that certain malignant cells can evade the immune response by targeting the expression of cGAS–STING components (or inhibiting their function), other evidence suggests that tumorigenesis is caused by chronic and enhanced STING activation. A pan‐cancer analysis of more than 10,000 samples from the TCGA database revealed that inactivating mutations and genomic loss of cGAS or STING occurred in less than 1% of tumor types. In addition, the expression levels of cGAS and TMEM173 (the STING gene) mRNA in many tumor tissues (such as breast, head, neck, lung, and pancreatic cancer) were increased (compared with levels in normal tissues).^236^ Therefore, the majority of tumors tend to retain cGAS and STING proteins (in favor of chronic inflammation), but tend to regulate downstream IFN signal transduction.

### 6.3 cGAS–STING signaling pathway in autoimmune disease

**FIGURE 6:**
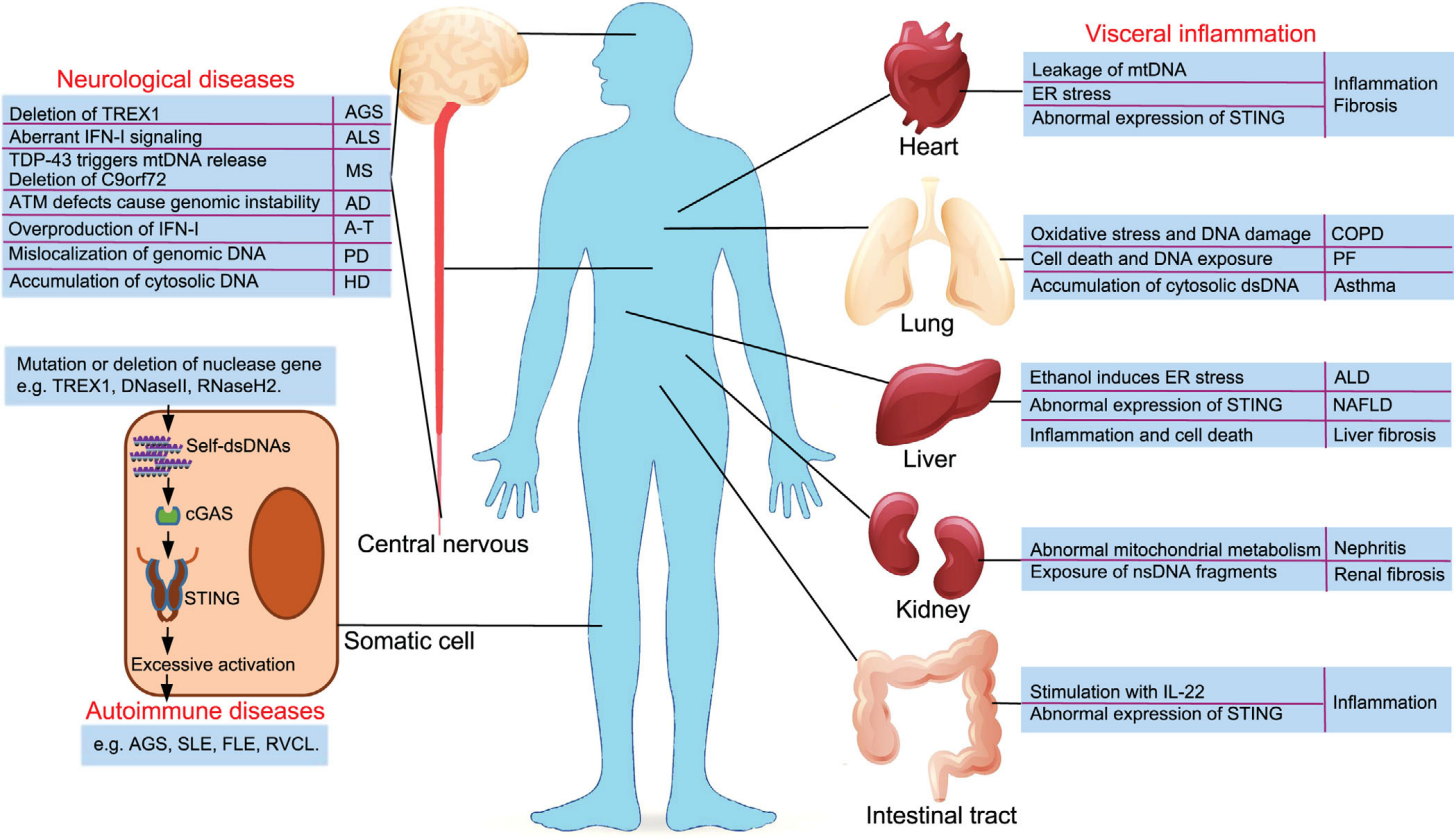
Activation of cGAS–STING signaling pathway in autoimmune diseases, neurological diseases, and visceral inflammations. Mutations in nucleases such as TREX1 (Three‐prime repair exonuclease), DNaseII (Deoxyribonuclease II), and RNaseH2 (Ribonuclease H2) lead to accumulation and exposure of self‐DNA in the cytoplasm, resulting in autoimmune diseases such as AGS (Aicardi‐Goutières syndrome), SLE (systemic lupus erythematosus), FLE (familial lupus erythematosus), and RVCL (retinal vasculopathy with cerebral leukodystrophy) (bottom left). Similarly, the activation of cGAS–STING signaling pathway in different causes leads to neurological diseases (top left), such as AGS, amyotrophic lateral sclerosis (ALS), multiple sclerosis (MS), ataxia‐telangiectasia (A‐T), Alzheimer's disease (AD), Parkinson's disease (PD), Huntington's disease (HD), and visceral inflammations (right), such as inflammation in the heart, lung, liver, kidney, and intestinal tract. ALD, alcoholic liver disease; COPD, chronic obstructive pulmonary disease; NAFLD, nonalcoholic fatty liver disease; PF, pulmonary fibrosis.

Activation of the cGAS–STING signaling pathway facilitates resistance to pathogens. However, improper or excessive activation may lead to serious abnormal inflammatory reactions, including autoimmune inflammation,^237^ which is associated with mutations in genes that regulate the cGAS–STING signaling pathway. For example, vascular and pulmonary syndrome is a systemic inflammatory disease caused by mutations in STING exons. STING functional mutation can significantly induce IFN‐β1 in vitro.^226^ In addition to such gene mutations, the lack of cytoplasmic nucleic acid exonuclease is another likely cause of autoimmunity. Under normal circumstances, endogenous nucleases in organisms are used to cut and remove unwanted cytoplasmic DNA. However, once these nucleases are deleted or mutated, exposure to self‐DNA is considerably increased, and the cGAS–STING signaling pathway is activated beyond the monitoring threshold, leading to the occurrence of autoimmune diseases (Figure 6).^8^

TREX1 is known to digest cytoplasmic DNA, including unmodified and oxidized DNA. *Trex1* ^−/‐^ mice show severe autoimmune phenotypes in multiple organs, with especially high expression levels of IFN and ISG in the heart. Mutation or deficiency of the TREX1 gene can cause a series of human autoimmune diseases, including Aicardi‐Goutières syndrome (AGS), systemic lupus erythematosus (SLE), familial lupus erythematosus (FLE), and retinal vasculopathy with cerebral leukodystrophy (RVCL).^238^, ^239^ Taking SLE as an example, the level of dsDNA in the serum of SLE patients is increased (compared with healthy people), and the membrane capsule derived from apoptotic cells in the serum induces ISG through the cGAS–STING signaling pathway. The expression level of the IFN‐induced gene IFIT3 in monocytes of SLE patients is also increased, and this interacts with STING and TBK1 to induce further activation of the cGAS–STING signal.^240^ In summary, cGAS–STING promotes the susceptibility and severity of autoimmune diseases and amplifies the autoimmune reaction of SLE.

Deoxyribonuclease II (DNase II) is an endonuclease located in the lysosome that eliminates exogenous DNA from dead cells. Animal studies have found that *DNase II* ^−/−^ mice die at the time of birth. Upon additional knockout of the cGAS or STING genes, *DNase II* ^−/−^ mice can not only develop normally (similar to wild‐type mice), and have no symptoms of arthritis. In addition, the level of anti‐dsDNA antibody is also significantly reduced.^241^ Therefore, deletion of DNase II also increases activation of the cGAS–STING signal, leading to systemic autoinflammation.^242^ However, to date, deletion of the human DNase II gene has not been reported.

Ribonuclease H2 (RNaseH2) removes ribonucleotides wrongly mixed with genomic DNA. The genome of *RNaseH2b* ^−/−^ cells is continuously unstable, which leads to the formation of micronuclei, the recruitment of cGAS, and ultimately activation of the IFN‐I cascade reaction.^78^, ^164^ Again, a cGAS or STING knockout can significantly reduce the inflammation and autoimmune phenotype of *RNaseH2* ^−/−^ mice.^85^, ^243^

In summary, the loss of the DNA clearance function in vivo leads to chronic activation of the cGAS–STING signaling pathway, and this then leads to autoimmune diseases.

### 6.4 cGAS–STING signaling pathway in neurological disease

The nervous system overlaps extensively with the immune system. Activation of the cGAS–STING signaling pathway by cytoplasmic DNA plays an important role in the occurrence and progression of several neurological diseases. Here, we focus on the latest discoveries concerning the cGAS–STING signaling pathway in neuroimmune diseases and neurodegenerative diseases (Figure 6).^244^

#### 6.4.1 Neuroimmune diseases

AGS is a life‐threatening hereditary encephalopathy similar to the innate immune response during viral infection caused by the abnormal induction of endogenous DNA. Mutation of a nuclease, such as exonuclease TREX1, in AGS patients results in the cellular accumulation of large amounts of self‐DNA. Thus, the pathogenesis of AGS is directly related to the assessed levels of cytoplasmic DNA, making cGAS a very attractive pharmacological target.^241^, ^244^

Multiple sclerosis (MS) is a chronic disease of the central nervous system, and it manifests as inflammatory demyelination. Recently, because of its important role of IFN‐I signal transduction in central nervous system inflammation, much attention has been given to the function of IFN‐I signal transduction in MS. As an important upstream key protein in IFN‐I signal transduction, STING has also become an important target for MS treatment.^245^ For example, the antiviral drug ganciclovir (GCV) inhibits the production of STING‐dependent IFN‐I to reduce neuroinflammation. However, GCV has no effect on STING‐deficient mice.^246^

Amyotrophic lateral sclerosis (ALS) is a motor neuron disease, and TARDNA‐binding protein 43 (TDP‐43), the major disease‐associated protein, is known to activate the cGAS–STING signaling pathway by triggering mtDNA release into the cytoplasm. Furthermore, deletion or inhibition of the STING gene improves neuroinflammation in ALS model mice.^69^ In another study, STING inhibition was shown to alleviate the overactive IFN‐I response in *C9orf72^−/−^* mice (which normally show STING‐IFN‐I‐mediated chronic inflammation).^247^ It can be concluded that cGAS–STING‐mediated inflammation promotes the progression of ALS.

#### 6.4.2 Neurodegenerative diseases

Ataxia‐telangiectasia (A‐T) is a multisystem disease caused by loss of activity of the protein kinase ataxia telangiectasia mutated (ATM) due to nonsense or missense mutations in the ATM gene. An accumulation of cytoplasmic DNA and a high level of IFN‐β expression were observed in Purkinje cells, microglia, and motor neurons from ATM‐deficient rats with neuroinflammation and a neurodegenerative phenotype. Further research revealed that genomic instability caused by ATM deficiency leads to the activation of cGAS–STING signal.^248^ Although these results provide evidence that activation of cGAS–STING signaling may be involved in A‐T cerebellar neurodegeneration, clinical data are required to support this result.

Alzheimer's disease (AD) is a neurodegenerative disease that develops when microglia in the brain activate their intracellular immune response and accumulate around amyloid beta (Aβ) plaques. During aging, DNA may become mislocated inside these microglial cells, activating the cGAS–STING molecular system and triggering inflammation.^249^ Neuroinflammation in AD is closely related to the production of IFN‐I mediated by excessive cGAS–STING signal. Indeed, loss of the IFN‐I receptor may prevent Aβ‐induced neurotoxicity, and inhibition of the cGAS–STING‐dependent IFN‐I response mitigates Aβ‐induced neuronal cell death and cognitive decline in AD.^250^, ^251^

Neuroinflammation is also an important feature of Parkinson's disease (PD), and cGAS–STING–IFN‐I signal transduction mediates neuroinflammation in PD pathology. Previous studies have provided evidence that IFN‐I signal is increased in post‐mortem brain tissue from PD patients, and that the production of IFN‐I promotes a neuroinflammatory reaction and disease progression in PD model mice. More importantly, cGAS–STING‐dependent IFN‐I signal transduction is a key regulator of the neuroinflammatory response and neuronal cell death in the early stage of PD.^252^ Furthermore, genomic DNA mislocation was also found to trigger cGAS–STING activation.^241^ It should also be noted that mitochondrial autophagy and mitochondrial DNA also trigger cGAS–STING‐mediated neuroinflammation.

Huntington's disease (HD) is an autosomal dominant genetic disorder and a neurodegenerative disease. However, the pathogenesis of HD is closely related to inflammation. Higher expression levels of proinflammatory cytokines such as IL‐6, IL‐8, and TNF‐α were observed in post‐mortem brain from HD patients.^253^, ^254^ In addition, cytoplasmic mtDNA is abundant in the tissues and cells of HD patients, triggering the cGAS–STING signaling pathway in the striatum.^255^, ^256^ Importantly, deletion of cGAS significantly inhibited the expression of INF‐I, indicating that the inflammatory response induced by cytoplasmic mtDNA in HD was mediated by the cGAS–STING signaling pathway.^257^ In other studies, cGAS was reported to upregulate and promote inflammation in HD, and depletion of cGAS was reported to reduce the expression of inflammatory genes in HD cells.^258^ Together, these findings demonstrate that inflammation mediated by cGAS–STING can help promote the pathological progression of HD, and they suggest that inhibition of cGAS–STING can help reduce inflammation‐related injury in HD.

In summary, cGAS–STING‐mediated IFN‐I signal transduction produces pleiotropic cytokines, and the cGAS–STING‐dependent IFN‐I response plays a central role in the occurrence and progression of neuroinflammation. Therefore, controlling the secretion of proinflammatory cytokines by targeting the cGAS–STING signaling pathway may help inhibit the occurrence and development of various neuroinflammatory diseases.

#### 6.4.3 Chronic pain

STING is a key regulator of nociception through IFN‐I signaling in peripheral nociceptors. Intrathecal activation of STING produces powerful nociceptive resistance in mice and non‐human primates.^259^ However, cGAS–STING may also be an important mediator of chronic pain. LPS‐induced polarization of M1 microglia is accompanied by activation of the cGAS–STING signaling pathway. cGAS and STING antagonists inhibit M1 polarization of microglia and improve mechanical abnormal pain induced by spared nerve injury (SNI).^260^ After exogenous and endogenous DNAs from pathogens and damaged cells activate the cGAS–STING signaling pathway, activation of STING promotes the expression of IFN‐I and NF‐κB, ultimately leading to chronic pain.^261^ In addition, the cGAS–STING signaling pathway activated by mtDNA leakage causes chronic postoperative pain by inducing IFN‐I and A1 reactive astrocytes in the spinal cord.^262^ Therefore, the cGAS–STING signaling pathway may play a bidirectional role in pain processing, regulating both antinociception and pronociception, although this proposal is still controversial.^263^

#### 6.4.4 Autism and depression

STING is also known to play a crucial role in the development of the cerebral cortex. Defects in dendrite morphogenesis lead to neuronal dysfunction and neurodevelopmental disorders, such as autism and depression. STING is essential for maintaining dendrite morphology, and *Sting* ^−/−^ mice exhibit autism‐like behaviors. Low doses of TNF‐α, an activator of NF‐κB, were observed to mitigate some of the autism‐like behaviors in *Sting* ^−/−^ mice.^264^ The chronic restraint stress (RST) mouse model exhibits depression‐like behavior, and increased levels of proinflammatory cytokines in the brain. The expression levels of STING, p‐TBK1, and p‐IRF3 were demonstrated to be significantly reduced in the hippocampus and prefrontal cortex of RST mice. However, 2′,3′‐cGAMP is able to activate the STING signaling pathway to inhibit the release of proinflammatory cytokines, including TNF‐α, IL‐6, and IL‐1β, in the brains of RST mice. The associated reduction in inflammation is accompanied by noticeable behavioral changes. Thus, the STING agonist induces antidepressant effects.^265^ Depression and anxiety are common neuropsychiatric symptoms of PD. In PD model mice, 1‐methyl‐4‐phenyl‐1,2,3,6‐tetrahydropyridine (MPTP) has been shown to induce depression‐like behavior, and this is accompanied by an increase in the expression levels of STING and IRF3 in the hippocampus. However, silibinin significantly attenuates MPTP‐induced depression/anxiety and downregulates the levels of IL‐1β, TNF‐α and IFN‐β, as well as the levels of STING and IRF3.^266^ These findings suggest that the cGAS–STING signaling pathway is associated with the development of depression and autism.

### 6.5 cGAS–STING signaling pathway in visceral inflammation

The cGAS–STING signaling pathway is closely related to inflammation and fibrosis in various visceral organs. Here, we review the correlation between inflammation of the liver, lung, kidney, intestine, heart, and other organs and the cGAS–STING signaling pathway (Figure 6).

#### 6.5.1 Liver

The cGAS–STING signaling pathway has been associated with numerous liver diseases. In addition to its association with hepatitis B and liver cancer, the cGAS–STING signaling pathway has been shown to be activated in alcoholic liver disease (ALD), nonalcoholic fatty liver disease (NAFLD), and liver fibrosis.

ALD is the most common type of chronic liver disease in the world. Studies have shown that the expression levels of IRF3, cGAS, TBK1, IKKε, and STING are positively correlated with the severity of ALD.^267^, ^268^ In alcohol‐administered mice, the expression levels of cGAS/STING pathway components were increased in the liver. Moreover, the effects of ALD were alleviated in mice lacking the cGAS or IRF3 genes. It was also reported that ethanol‐induced ER stress triggered the interaction between IRF3 and the ER adapter STING. Conclusively, deletion of STING inhibited ethanol‐ or ER stress‐induced phosphorylation of IRF3, while deletion of IRF3 prevented hepatocyte apoptosis and reduced liver injury.^269^ In conclusion, cGAS–STING signal activation may be important in the pathogenesis of ALD, and targeting the cGAS–STING signaling pathway is expected to become a therapeutic strategy for ALD.

NAFLD is one of the world's most important causes of liver disease. Research has revealed that STING protein levels in macrophages and endothelial cells obtained from the liver tissues of NAFLD patients were higher than those of non‐NAFLD patients.^270^, ^271^ Furthermore, animal studies provide evidence that the cGAS–STING signaling pathway is involved in the development of NAFLD. The expression levels of STING and its downstream factor IRF3 were significantly increased in the livers of mice fed a high‐fat diet (HFD). Moreover, the STING protein changes were found to be specific to bone marrow cells, and these changes were observed to aggravate HFD‐induced NAFLD. Critically, mice with a STING knockout and damaged bone marrow cells exhibited less hepatic steatosis and less inflammation.^270^, ^271^ Thus, cGAS–STING activation plays a significant role in the pathogenesis and progression of NAFLD.

Liver inflammation often accompanies the formation of liver fibrosis. In some cases of steatohepatitis, the deposition of fat in hepatocytes can activate the inflammatory response triggered by the STING signaling pathway,^272^ and this inflammatory response further activates hematopoietic stem cells (HSCs). Through cell‐to‐cell interactions and signal transduction, the HSCs are transformed into myofibroblast‐like cells, which in turn secrete α‐SMA and hepatocyte growth factor to accelerate liver fibrosis.^270^, ^273^, ^274^ Furthermore, when WT mouse hepatocytes were treated with ccl4, ER stress activated the STING signaling pathway, resulting in extensive phosphorylation of TBK1 and IRF3, increased hepatocyte apoptosis, and liver fibrosis. These changes were not observed when the experiment was repeated with STING‐deficient mice.^275^ STING expression level in monocyte‐derived macrophages has also been implicated in the progression of liver inflammation and fibrosis in NAFLD.^276^ In addition, activation of STING signal is known to trigger liver inflammatory responses, to exacerbate the impairment of liver sinusoidal endothelial cells (LSECs), and to increase the contribution of hepatic sinusoidal microthrombosis to liver fibrosis.^277^

#### 6.5.2 Lung

Many lung diseases demonstrate an association with inflammation and excessive autoimmune responses. In this context, the cGAS–STING signaling pathway has been widely studied because of its importance in the occurrence of lung inflammatory diseases,^271^ such as chronic obstructive pulmonary disease (COPD), pulmonary fibrosis (PF), and asthma.

COPD is characterized by chronic and long‐term lung inflammation, often resulting from oxidative stress and DNA damage caused by the excessive production of ROS. The long‐term inflammatory response in COPD is linked to activation of the cGAS–STING signaling pathway by damaged DNA. In mice, cigarette smoke‐induced damage to the mucous membranes of the respiratory tract results in the release of DNA into the cytoplasm, thus activating the cGAS–STING signaling pathway and triggering IFN‐I‐dependent lung inflammation. Critically, lung inflammation was significantly reduced in a STING gene knockout mouse model.^278^

In addition to participating in COPD, the cGAS–STING signaling pathway also plays an important role in PF. For example, STING has been shown to play a significant role in silica‐induced lung disease. In patients with silicosis, plasma levels of dsDNA were significantly higher than those of normal subjects, and the cGAS–STING signaling pathway was activated in lung cells of patients exposed to silica particles.^279^ Animal experiments revealed that silica particles triggered lung cell death, and the release of dead cell dsDNA into the bronchoalveolar space subsequently increased STING expression level and activation. However, the expression level of STING was decreased in patients with acute exacerbation of idiopathic fibrosis and in mouse models of PF. After treatment, the expression levels of STING were significantly increased in PF patients.^280^ Indeed, low levels of STING expression were associated with an increase in collagen deposition and fibrosis in the lung, indicating that STING has an inhibitory effect on idiopathic PF.^281^ In summary, STING appears to play different roles in PF, and a full understanding of these roles requires further experimental research and verification.

Asthma is a chronic inflammatory lung disease characterized by hyperresponsiveness and reversible impairment of airway ventilation. In mouse asthma models generated by a challenge with chicken ovalbumin or dust mites, an accumulation of dsDNA in the cytoplasm of mouse airway endothelial cells leads to allergic airway inflammation. Importantly, the absence of cGAS significantly reduced this inflammatory response.^282^ Therefore, activation of the cGAS–STING signaling pathway may be responsible for the immune response observed during asthma attacks. However, further research is needed to confirm this hypothesis. In addition, ISG expression levels in patients with mild and severe asthma were reportedly increased, and increased the levels of ISG expression in the airway were positively correlated with decline in lung function. More importantly, there may be a potential link between ISG‐mediated inflammation and ER stress.^283^

In conclusion, additional research is urgently required to confirm the role of the cGAS–STING signaling pathway in the pathogenesis and development of various pulmonary inflammatory diseases, and the influence of cytokines mediated by the cGAS–STING signaling pathway on patients with lung diseases should be clarified.

#### 6.5.3 Kidney

The cGAS–STING signaling pathway may also be associated with acute or chronic kidney disease (CKD).^284^ In a study on lupus nephritis (LN), TNF‐induced apoptosis was demonstrated to produce IFN‐I, potentially explaining the associated kidney injury.^285^ In another study, cisplatin‐induced acute kidney injury in mice was shown to be caused by cGAS–STING signaling pathway activation via mtDNA, ultimately leading to inflammatory damage. Importantly, renal inflammation was not observed in STING knockout mice, confirming the important role of cGAS–STING activation in acute kidney inflammation.^286^ Recent studies have found that the cGAS–STING signaling pathway is activated during the pathogenesis of CKD, and activation of this pathway is related to mitochondrial metabolism abnormalities. In a CKD model generated using STING knockout mice, renal failure and fibrosis were significantly suppressed following STING deletion.^287^ Together, the above results demonstrate the contribution of the cGAS–STING signaling pathway to the occurrence and development of CKD. In addition, nucleosomal‐associated dsDNA (nsDNA) fragments have been shown to trigger activation of the cGAS–STING signaling pathway, and to lead to the expression of IFN‐β, further aggravating renal inflammation in LN‐associated end‐stage renal disease.^288^

Renal fibrosis is the final stage in the various signaling pathways leading to end‐stage renal failure. In mitochondrial transcription factor A (TFAM) gene knockout mice, mtDNA is released into the cytoplasm of renal cells to activate the cytoplasmic cGAS–STING signaling pathway, leading to cytokine transcription and immune cell recruitment. The cGAS–STING signaling pathway was shown to regulate 6‐phosphofructo‐2‐kinase/fructose‐2,6‐bisphosphatase 3 (PFKFB3)‐mediated glycolysis by promoting the phosphorylation of IRF3, which in turn played a driving role in hypoxia‐induced renal fibrosis.^289^ Critically, the deletion or inhibition of STING significantly improved renal fibrosis in a mouse model of CKD.^287^

In conclusion, the above findings provide evidence for the important role of the cGAS–STING signaling pathway in acute/chronic renal diseases. In animal models, renal inflammatory damage can be alleviated by knocking out key genes in the cGAS–STING signaling pathway, which indicates that precise intervention at targets in the cGAS–STING signaling pathway may be beneficial in treating kidney‐related diseases.

#### 6.5.4 Intestine

The cGAS–STING signaling pathway is also known to play an important role in intestinal inflammation. In a mouse model of colitis induced in wild‐type mouse by administering dextran sodium sulfate (DSS), the expression level of STING protein was increased, and treatment with a STING agonist significantly aggravated the disease. Crucially, the signs of colitis were significantly improved in STING gene knockout mice.^290^ These findings suggest that activation of the STING signaling pathway may contribute to the progression of intestinal inflammation. Furthermore, IL‐22 is reported to induce high level expression of ISG through the cGAS–STING signaling pathway, and this is known to determine the severity of intestinal inflammation.^291^ The STING signaling pathway has also been shown to be crucial to intestinal homeostasis. For example, symbiotic bacteria stimulate the STING signaling pathway to produce proinflammatory and anti‐inflammatory cytokines. A study also revealed that the level of IL‐22 mRNA in the colon of mice with anti‐inflammatory cytokine IL‐10 deficiency was significantly increased.^292^ However, whether IL‐10 can negatively regulate IL‐22 to achieve negative feedback regulation of the cGAS–STING signaling pathway remains to be determined. In additional research, inhibition of the cGAS–STING signaling pathway by atrial natriuretic peptide (ANP) was observed to alleviate DSS‐induced colitis.^293^ Moreover, the survival of wild‐type mice was significantly improved after treatment of DSS‐induced colitis with the cGAS inhibitor RU.521. Treatment with RU.521 was also shown to reduce cGAMP levels during colitis, and to reduce the phosphorylation levels of STING, TBK1, and IRF3. Together, these results reveal the potential pathogenic role of the cGAS–STING signaling pathway in colitis.^294^

In conclusion, the above studies provide evidence that hyperactive cGAS–STING signal can promote intestinal inflammation. However, further research is needed to clarify whether the excessive activation of cGAS–STING signal is related to cytoplasmic DNA or microbial DNA.

#### 6.5.5 Heart

Heart disease is one of the most common causes of death worldwide. Inflammation, fibrosis, and oxidative stress can lead to heart‐related diseases. Recent studies have found that the cGAS–STING signaling pathway plays an important role in the pathology of various heart diseases.^271^, ^295^ For example, the expression levels of cGAS–STING signaling pathway‐related genes in the hearts of SLE patients were significantly higher than those in the hearts of normal subjects.^296^ Another study found that the cGAS–STING signaling pathway aggravated the proinflammatory response in a mouse model of myocardial infarction.^297^ After deletion of cGAS or STING, myocardial function and the survival rate in myocardial infarction mice were both improved.^61^ In addition, high expression levels of genes related to the cGAS–STING signaling pathway have been associated with cardiac insufficiency (irrespective of the causative factors), and when the cGAS–STING signaling pathway is inhibited, cardiac function is significantly restored.^271^

The cGAS–STING signaling pathway may also play an important role in cardiac dysfunction caused by cardiac structural abnormalities. STING expression levels were reported to be increased in patients with dilated cardiomyopathy (DCM) and hypertrophic cardiomyopathy (HCM). When STING was inhibited, the cardiac hypertrophy and fibrosis of DCM and HCM were significantly reduced.^298^ Moreover, in a heart failure model induced by nonischemic pressure overload, cardiac dysfunction, fibrosis, and IFN‐I expression levels were all increased. Conversely, cardiac function and IFN‐I expression levels returned to normal. In addition, when treated with angiotensin II, the expression levels of STING and the IFN‐I expression levels were normal in STING gene knockout mice. Finally, the expression levels of STING and IFN‐I in neonatal rat cardiac myocytes were increased after treatment with angiotensin II, while the expression levels of proinflammatory factors and IFN‐I in these cells were significantly decreased after inhibition of STING expression by siRNA.^299^, ^300^

The cGAS–STING signaling pathway mechanism in the heart has already been elucidated. Research has shown that palmitic acid (PA) activates the cGAS–STING signaling pathway in endothelial cells by causing mitochondrial damage and inducing release of mtDNA into the cytoplasm.^297^ Similarly, leakage of mtDNA into the cytoplasm may be an important factor in cGAS–STING signaling pathway activation in the heart. Activation of the cGAS–STING signaling pathway by ER stress is thought to be a key mechanism in the progression of heart disease. In in vitro experiments, an ER stress activator significantly increased the expression level of STING. Importantly, inhibition of STING in a mouse model of myocardial hypertrophy significantly reduced both hypertrophy and fibrosis.^298^ These findings provide evidence that STING expression level is significantly related to ER stress, and that high level expression of STING may promote the development of cardiac hypertrophy. In addition to the above mechanisms, the cGAS–STING signaling pathway was reported to play a role in determining the polarity of macrophages in a mouse model of myocardial infarction. In the obesity‐related diabetic cardiomyopathy mouse model, lipotoxicity‐induced mtDNA release was observed to induce cardiac cell death and fibrosis via activation of the cGAS–STING signaling pathway and subsequent inflammation.^301^

In conclusion, the cGAS–STING signaling pathway plays a significant role in aggravating heart disease. However, the underlying mechanism is very complex, and in‐depth research is needed to clarify the role of the cGAS–STING signaling pathway in heart disease progression.

### 6.6 Others

Obesity is a global epidemic caused by excessive energy intake or inefficient energy consumption. In a mouse model of mitochondrial stress in adipose tissue, a fat‐specific knockout of disulfide‐bond A oxidoreductase‐like protein (DsbA‐L) was found to cause mitochondrial damage and promote mtDNA release, resulting in cGAS–STING signaling pathway activation and an inflammatory response. Conversely, adipose‐specific overexpression of DsbA‐L protected mice from cGAS–STING signaling pathway activation and subsequent inflammation caused by a high‐fat diet.^300^, ^302^ The cGAS–STING signaling pathway activated by mitochondrial stress acts as a warning signal to inhibit heat production in adipose tissue. Therefore, targeting the adipose cGAS–STING signaling pathway may be a potential treatment strategy for obesity and related metabolic diseases caused by excess nutrition.

Diabetes is a metabolic disease that causes multiple organ damage. In general, diabetes is induced by an absolute or relative insulin deficiency. Studies have shown that inflammation can lead to obesity, insulin resistance (IR), and the serious complications associated with advanced diabetes.^271^ However, the exact relationship between obesity and inflammation remains to be fully clarified. Knockout of the downstream targets of cGAS–STING, TBK1, or IRF3^303^, ^304^ or inhibition of IκB kinase ε (IKKε) and TBK1 with amlexanox, resulted in significant weight loss, enhanced insulin sensitivity, and improved glucose tolerance in obese mice (and some type II diabetic patients).^305^, ^306^ However, the exact role of the cGAS–STING signaling pathway in IR and energy consumption during cellular metabolic processes remains to be elucidated. The cGAS–STING signaling pathway was also reported to be associated with diabetes through Akt and DsbA‐L, but the study was only a correlation analysis, and no definitive evidence has been found.^271^ It can be concluded that in‐depth research is now necessary to understand the relationship between the cGAS–STING signaling pathway and the pathogenesis of diabetes.

Dysfunction of the cGAS–STING signaling pathway may also play a role in various cutaneous diseases.^307^ It has been reported that expression levels of STING and its downstream factors are significantly higher in diseased skin samples from psoriasis patients (compared with levels in skin samples from healthy people), These results reveal the potential involvement of STING dysregulation in the pathogenesis of psoriasis. The STING agonist 5,6‐dimethylxanthone‐4‐acetic acid (DMXAA) is known to aggravate psoriasis symptoms and skin inflammation, while the STING antagonist H‐151 exhibits anti‐inflammatory activity in imiquimod (IMQ)‐induced psoriasis mice,^308^, ^309^ it can be concluded from these results that STING activation promotes the occurrence of psoriasis inflammation. In addition, melanoma cell‐derived DNA induces STING and IRF3 signal transduction in dendritic cells to trigger IFN‐β production. However, the cGAS–STING pathway is inhibited in human skin cutaneous melanoma, resulting in reduced melanoma IFN‐I production and loss of tumor antigenicity, characteristics that are essential for melanoma immune evasion.^310^ Thus, melanoma‐specific STING inhibition leads to reduced IFN‐I production and immunosuppression in melanoma, revealing a novel STING dysfunction mechanism in skin cutaneous melanoma.

## 7 TARGETTING cGAS–STING SIGNALING PATHWAY FOR DISEASE THERAPY

The activation and inhibition of the cGAS–STING signaling pathway in various disease states are discussed above. Based on these studies, researchers have developed a series of agonists and inhibitors of the cGAS–STING signaling pathway. Here, we summarize the known inhibitors and agonists of the cGAS–STING signaling pathway, and we note their drug regimens and delivery methods.

### 7.1 Inhibitors of the cGAS–STING signaling pathway

With key molecules in the cGAS–STING signaling pathway as the intervention target, drugs are screened or rationally designed to inhibit activation of the cGAS–STING signal to treat associated diseases. To date, research on cGAS–STING inhibitors has mainly focused on the preclinical stage. Here, we summarize the research progresses on small molecule drugs targeting DNA, cGAS, STING, and TBK1 (Table 2).

**TABLE 2:** Targets, structures, and mechanisms, and IC50 of inhibitors for the cGAS–STING signaling pathway.

Target	Inhibitor	Structure	Mechanism	IC50 (μM)	References
DNA	Hydroxychloroquine	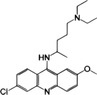	Blocking the cGAS–dsDNA	23	^311^
DNA	Quinacrine	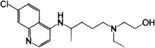	Blocking the cGAS–dsDNA	7	^312^
DNA	X6	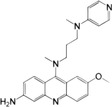	Blocking the cGAS–dsDNA	13	^311^
cGAS	Suramin	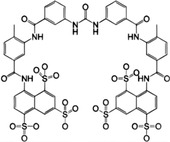	Competing with dsDNA	<5	^316^
cGAS	PF‐06928215	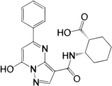	Inhibiting the catalytic activity	4.9	^317^
cGAS	RU.365	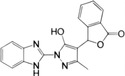	Inhibiting the catalytic activity	4.98	^318^
cGAS	RU.521	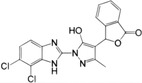	Inhibiting the catalytic activity	0.7	^318^
cGAS	G150	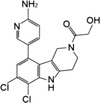	Inhibiting the catalytic activity	0.0102	^319^
cGAS	Compound S3	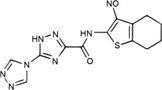	Inhibiting the catalytic activity	4.9	^320^
cGAS	CU‐76	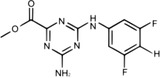	Unknown	0.24	^321^
STING	Tetrahydroisoquinolines Compound 1	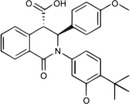	Competing with CDN	7.3	^322^
STING	Tetrahydroisoquinolines Compound 18	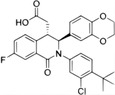	Competing with CDN	11	^322^
STING	Astin C	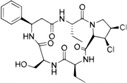	Competing with CDN	10.83	^323^, ^324^, ^325^
STING	Nitrofurans C‐176	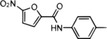	Inhibiting the palmitoylation	Unspecified	^99^
STING	Nitrofurans C178	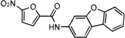	Inhibiting the palmitoylation	Unspecified	
STING	Nitrofurans C‐170	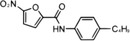	Inhibiting the palmitoylation	Unspecified	
STING	Nitrofurans C‐171	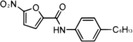	Inhibiting the palmitoylation	Unspecified	
STING	Nitro fatty acids (NO_2_‐cLA)	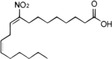	Inhibiting the palmitoylation	Unspecified	^158^
STING	Nitro fatty acids (NO_2_‐OA)	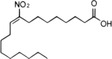	Inhibiting the palmitoylation	Unspecified	
STING	Indole ureas (H‐151)	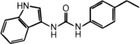	Inhibiting the palmitoylation	Unspecified	^99^
STING	Acrylamides (BPK‐21)	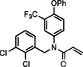	Inhibiting the palmitoylation	Unspecified	^326^
STING	Acrylamides (BPK‐25)	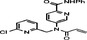	Inhibiting the palmitoylation	Unspecified	
STING	Butenolide heterodimer Compound 13	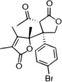	Unknown	Unspecified	^327^
TBK1	BX795	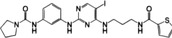	Competing with ATP	0.006	^328^, ^329^
TBK1	MRT67307	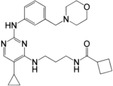	Selectively inhibiting TBK1 activity	0.019	^330^, ^331^
TBK1	Tozasertib‐15a	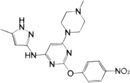	Inhibiting Ser/Thr kinase or TBK1	0.06	^332^
TBK1	Pyrazolopyrimidine derivative Compound II	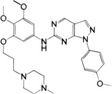	Impairing Akt signaling	0.013	^332^, ^333^
TBK1	Amlexanox	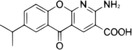	Directly inhibiting TBK1	1‐2	^334^, ^335^
TBK1	Azabenzimidazole HIT 1a	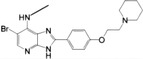	Directly inhibiting TBK1	0.151	^341^
TBK1	AZ13102909	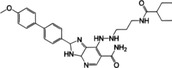	Directly inhibiting TBK1	0.005	^342^
TBK1	Idronoxil	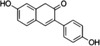	Inhibiting the phosphorylation	0.96	^340^

Abbreviations: Akt, protein kinase B; CDN, cyclic dinucleotide; dsDNA, double‐stranded deoxyribonucleic acid; NO_2_‐cLA, nitro‐linoleic acid; NO_2_‐OA, nitro‐oleic acid.

#### 7.1.1 Inhibitors targeting DNA binding

##### Antimalarial drugs

Some antimalarial drugs, such as hydroxychloroquine and quinacrine, have been used as potential treatments for SLE, and these inhibit IFN‐β production by binding to DNA and selectively blocking cGAS–dsDNA interactions. The second‐generation molecule X6 has been shown to inhibit the binding of cGAS to dsDNA in vitro and in vivo in a stable and effective interaction.^311^ A study of the underlying mechanism reveals that X6 binds to the small groove of DNA via interactions involving positively charged amino side chains, indirectly inhibiting cGAS activation by binding the dsDNA and preventing the assembly of the cGAS–dsDNA complex.^312^

##### Inhibitory oligodeoxynucleotides

A151 is an inhibitory oligodeoxynucleotide containing four TTAGGG repeat motifs (5′‐tt agg gtt agg gtt agg gtt agg g‐3′). A151 was found to bind to the dsDNA‐binding domain of cGAS as a competitive inhibitor of DNA.^313^ However, the exact binding site of A151 in the dsDNA binding domain needs to be clarified. In addition, A151 was also able to inhibit the production of IFN‐I in TREX1‐deficient cells, raising the possibility that A151 may be used for the treatment of dsDNA‐mediated autoimmune diseases.^313^

#### 7.1.2 cGAS inhibitor

A number of studies have reported the rational design of novel cGAS inhibitors. In general, cGAS antagonists bind one of the cGAS active sites, competing against either the ATP or GTP substrates or the cGAMP product. Several other cGAS antagonists bind to cGAS and shield the dsDNA binding site (acting as competitive inhibitors with DNA), thus interfering with the initial activation of cGAS.^65^, ^311^, ^312^, ^314^, ^315^, ^316^

##### Shield DNA‐binding sites on cGAS

Suramin was identified as an inhibitor of cGAS activity by screening. The experimental data suggest that suramin inhibits the activity of cGAS by binding to the dsDNA‐binding site on cGAS, thus inhibiting assembly of the cGAS–dsDNA complex. Because this inhibitory activity is cGAS‐specific, suramin inhibition does not affect the TLR1, TLR2, or TLR4 pathways. Although suramin also affects cell activity, the anionic sulfate of suramin has been developed as a similar that also binds to the dsDNA‐binding region on cGAS.^316^

##### cGAS active site inhibitor

PF‐06928125 is a pyrazole pyrimidine compound screened for cGAS binding. X‐ray crystallography was used to confirm that this compound combines with the active site of cGAS, and PF‐06928125 was observed to compete with the adenosine base of ATP or cGAMP. While this compound has been demonstrated to bind cGAS in biochemical assays, PF‐06928125 lacks inhibitory activity in cell assays.^317^ It is speculated that the content of ATP and GTP in cells is too high, so more effective compounds may be needed to inhibit cGAS at these active sites.

In another study, compound RU.365 was identified from a library containing 123,306 compounds by high‐throughput screening of cGAS inhibitors. A crystal structure of the mouse cGAS, dsDNA, and RU.365 ternary complex revealed that RU.365 was bound to only one side of the pocket of the active site of cGAS, which is similar to the binding interaction between cGAMP and cGAS. RU.521 is an analog of RU.365 that occupies more space in the active site pocket of cGAS (compared with RU.365). Moreover, RU.521 exhibits better binding affinity with mouse cGAS (compared with RU.365), and enhanced inhibition.^318^

Recently, a compound with pyridine indole as the core was screened for cGAS binding. This compound, G150, represents a new class of cGAS active site‐targeting inhibitor. When tested in THP‐1 and human primary macrophages, G150 inhibition of cGAS proved very effective, as judged by detection of downstream IFN‐β1 mRNA levels. In addition, after a series of tests to determine its effect on other innate immune signaling pathways, G150 was demonstrated to selectively inhibit the cGAS–STING signaling pathway. An X‐ray structure of the cGAS–G150 complex confirmed that G150 bound the active site of cGAS.^319^

Another identified compound, S3, exhibits an IC_50_ of 4.9 μM. While the binding mode of compound S3 has been compared with those of PF‐06928125 and RU.521, no other research data have been published to date.^320^

##### Compounds with unknown mechanism

Recently, researchers conducted a high‐throughput screening of a large drug library in silico using the protein–protein interface from the cGAS/dsDNA complex to identify new small molecule inhibitors of hcGAS. After a series of optimizations, the authors were able to identify and validate CU‐76 as an effective inhibitor of hcGAS. While CU‐76 selectively inhibited the DNA‐activated signaling pathway in human cells, it did not affect the RIG‐I‐MAVS or Toll‐like receptor signaling pathways. However, the researchers could not obtain the crystal structure of CU‐76 combined with cGAS or determine its specific inhibition mechanism.^321^ Nevertheless, the small drug molecules identified provide a new chemical structure basis for developing hcGAS inhibitors with potential therapeutic applications. Moreover, they serve as an effective small molecule chemical probe for studying the biological function and related diseases of cGAS in human cells.

#### 7.1.3 STING inhibitor

To date, the inhibition mode of all STING inhibitors has been based on one of two mechanisms. First, by occupying the binding site for CDN, the inhibitor acts as a competitive antagonist of STING ligands. Second, by binding with the Cys88 or Cys91 residues near the STING protein TM domain, thus targeting the STING palmitoylation site. In the following chapters, we consider these two inhibitor classes, briefly introducing the inhibitor design and then describing representative STING inhibitors.

##### STING antagonists shielding CDN binding sites

Biophysical and X‐ray crystallographic data reveal that the C‐terminal domain of STING exists in the form of a symmetric dimer, with ligand binding sites at the interface between monomers. To exploit this, small molecule antagonists that bind to the CDN binding sites were designed based on the symmetry of the CDN binding domains. Tetrahydropisoquinoline Compound 1 was identified using ligand screening technology based on mass spectrometry. Considering the molecular conformation of STING, Compound 1 was modified to obtain Compound 18, and this was demonstrated to bind STING in a way that shields the CDN binding site. To validate its activity, Compound 18 was used to inhibit cGAMP‐induced IFN‐β secretion in THP‐1 cells.^322^

The natural product astin was identified as an inhibitor of STING in a cyclic peptide library screening using a reporter gene. A pull‐down experiment using biotinylated astin C and human STING was then used to demonstrate that astin C competitively binds to CDN sites. When excessive unlabeled free astin C was added, the binding of biotinylated astin C with STING was significantly decreased. Mechanistic studies revealed that astin C blocked the recruitment of IRF3 by STING, thus preventing downstream signal transduction. In an in vitro study, astin C was observed to inhibit the expression of IFN‐I in *Trex1* ^−/−^ BMDMs.^323^ In an in vivo study, astin C was observed to strongly inhibit the mRNA expression levels of *Ifn‐b*, *Cxcl10*, *Isg15*, *Isg56*, and *Tnf* in the heart, muscle, stomach, and tongue of *Trex1^−/−^* mice. Through these studies, the in vivo inhibitory effect of astin C on STING has been conclusively demonstrated.^324^, ^325^

##### Targeting the STING palmitoyl sites

A series of nitrofurans were screened using HEK293T cells overexpressing mouse STING. Protein mass spectrometry revealed that these inhibitors specifically bind Cys91 of STING (but not Cys88). Of the nitrofuran compounds identified, C‐176 was observed to bind STING with strong selectivity. Pretreatment of mice with C‐176 significantly reduced the STING agonist‐mediated increase in serum levels of IFN‐I and IL‐6. In addition, treatment with C‐176 significantly reduced serum IFN‐I levels in *Trex1* ^−/−^ mice, and it also strongly suppressed cardiac inflammation. Another compound, C‐178, with similar action and effect to C‐176 was identified, and the activities of these compounds were demonstrated to be closely related to the nitro and furan groups. While compounds C‐176 and C‐178 specifically bind mouse STING only, compounds with butyl (C‐170) or hexyl (C‐171) groups at the fourth position of the phenyl ring exhibited inhibitory activity against STING in both humans and mice.^99^

Endogenous nitro fatty acids (NO_2_‐FA), such as nitro‐linoleic acid (NO_2_‐cLA) and nitro‐oleic acid (NO_2_‐OA), covalently modify STING through a Michael addition reaction. NO_2_‐FA treatment of THP‐1 cells and BMDMs infected with HSV‐2 was observed to reduce intracellular IFN‐I production. The study of the mechanism revealed that NO_2_‐FA induced alkylation at Cys88, Cys9, and His16 of STING (in a nonselective manner). In addition, when NO_2_‐FA was used to treat fibroblasts from patients with STING‐related infantile vascular disease (SAVI), intracellular hyperphosphorylation of TBK1 was significantly inhibited.^158^

Another kind of indole urasse, H‐151, was successfully screened using HEK293T cells overexpressing mouse STING. Similar to the nitrofurans described above, protein mass spectrometry revealed that H‐151 also formed a covalent bond with Cys91 of STING. Moreover, H‐151 exhibited inhibitory activity against both human and mouse STING. In in vitro experiments, H‐151 was observed to inhibit IFN‐I production and TBK1 phosphorylation in various cells. In animal experiments, the STING agonist‐mediated cytokine response was significantly inhibited after H‐151 was administered intraperitoneally. In addition, after 1 week treatment of Trex1^−/−^ mice with H‐151, IFN‐β levels in vivo were significantly reduced.^99^

Mass spectrum analysis was used to demonstrate that the arylamides BPK‐21 and BPK‐25 combined with STING Cys91 to form an adduct. In addition, experiments revealed that BPK‐25 inhibited the cGAMP–STING signaling pathway in peripheral blood monocytes.^326^

##### Inhibitors with unknown mechanism

In a screen of 250,000 compounds for cGAS–STING signaling pathway inhibitors based on their effect on cell phenotype, researchers identified butenolide heterodimer Compound 13. After treatment with Compound 13, the dsDNA‐mediated increase in CXCL10 mRNA level in THP‐1 cells was significantly reduced, indicating that the IFN‐I signal was reduced.^327^ Although Compound 13 has been proven to regulate the cGAS–STING signaling pathway, its exact mechanism of action is still unclear.

#### 7.1.4 TBK1 inhibitor

##### BX795 and pyrimidine derivatives

BX795 is an effective inhibitor of the TBK1/IKKε signaling pathway. BX795 was initially found to be an inhibitor of phosphoinositol‐dependent kinase 1 (PDK1) and an ATP‐competitive inhibitor of TBK1. Treatment of primary peripheral blood mononuclear cells (PBMCs) with BX795 was observed to inhibit the phosphorylation of IRF3 and the production of IFN‐I.^328^ BX795 was also reported to inhibit IFN‐α production in PBMCs from patients with STING‐related diseases. In addition, BX795 reduced STAT1 phosphorylation and ISG mRNA expression.^329^ Another aminopyrimidine compound similar to BX795, MRT67307, inhibited TBK1 more selectively, and did not significantly inhibit the kinases IKKα/β, JAK, and p38 MAPK.^330^, ^331^ The pyrimidine compound tozasertib‐15a and the pyrazolopyrimidine derivative Amgen compound II have also both been identified as TBK1 inhibitors.^332^, ^333^

##### Amlexanox and its derivatives

Amlexanox is an anti‐inflammatory and antiallergic drug used to treat oral ulcers and asthma. In a mouse model, Amlexanox was demonstrated to alleviate hepatic fibrosis and acute injury induced by acetaminophen by inhibiting TBK1/IKKε.^334^, ^335^ However, the low solubility and medium potency of Amlexanox limit its further application. To address this problem, the C3‐carboxylic acid and C7‐isopropyl groups of Amlexanox were modified to produce analogs. While tetrazolium‐substituted Compound 21 demonstrated a strong inhibitory effect on TBK1 and IKKε, the activity of this compound in cells was low. Among other analogs, Compound 22, a C7‐cyclohexyl analog, was demonstrated to inhibit IL‐6 secretion in 3T3‐L1 cells, although further studies revealed limitations in applying each of these compounds.^336^, ^337^, ^338^, ^339^

##### Other small molecule TBK1 inhibitors

Idronoxil (IDX), a synthetic flavonoid closely related to daidzein, was recently demonstrated to effectively inhibit STING signaling. Specifically, IDX prevented the formation of the TBK1 and STING complex, blocking the phosphorylation of TBK1 at Ser172, and ultimately preventing the nucleosomal transcription of IRF3 and NF‐κB. Notably, IDX exhibited significant efficacy in a model devised for the treatment of COVID‐19. Thus, IDX is a potential therapeutic drug for the treatment of a broad range of inflammatory diseases.^340^ In addition, the azabenzimidazole derivatives HIT 1a, and AZ13102909 have also been confirmed to inhibit TBK1, but further experimental studies are warranted.^341^, ^342^

Finally, it should be noted that long‐term suppression of TBK1 may increase the risk of viral infection, and caution should be exercised with these immunomodulatory compounds.

### 7.2 Agonists of the cGAS–STING signaling pathway

**FIGURE 7:**
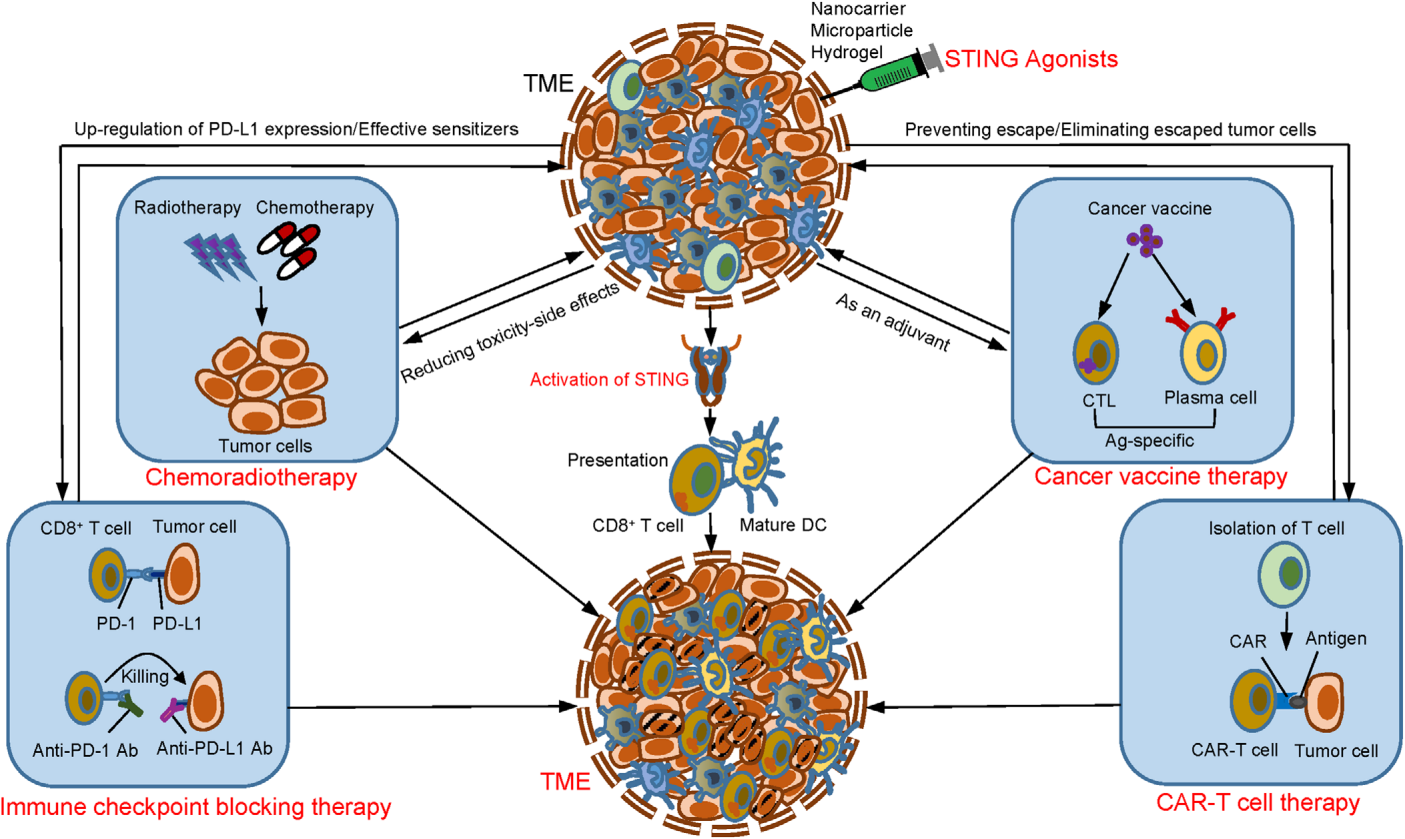
STING agonists alone and in combination for cancer therapy. Using nanocarrier, microparticle, or hydrogel as carrier, STING agonists (red font) enter the TME (tumor microenvironment) and directly activate the STING‐dependent antitumor immune response to eliminate tumor cells. In addition, STING agonists can also be combined with chemoradiotherapy, immune checkpoint blocking therapy, cancer vaccine therapy, and CAR‐T cell therapy (red font) to seek better antitumor effects. CAR‐T, chimeric antigen receptor‐modified T cells; PD‐1/PD‐L1, programmed death receptor 1/programmed death ligand 1.

Activation of the cGAS–STING signaling pathway in the TME can trigger specific monitoring of tumor Ags and promote effector T cells to infiltrate tumor tissue. Given the immunostimulatory potential of the cGAS–STING signaling pathway, development of STING agonists and their application are of the utmost relevance in cancer therapy. STING agonists include CDN analogs and their derivatives, DMXAA and its analogs, indirect agonists, and small molecule agonists (Figure 7 and Table 3).

**TABLE 3:** Classifications, application models, trial phases, and statuses of STING agonists.

Agonist	Classification	Application model	Phase (Trial Number)	Status	References
c‐di‐GMP	Natural CDN	Colon cancer	Preclinical	−	^344^
		Breast cancer			
3',3'‐cGAMP	Natural CDN	Chronic lymphocytic leukemia	Preclinical	−	^345^
2',3'‐cGAMP	Natural CDN	Colon cancer	Preclinical	−	^347^, ^348^
		Breast cancer			
		Squamous cell carcinoma			
		Melanoma			
ADU‐S100	Synthetic CDN	Advanced solid tumors;	I (NCT02675439)	Terminated	^350^, ^351^, ^352^
		Lymphomas; Head and neck cancer	II (NCT03937141)	Terminated	
IACS‐8779	Synthetic CDN	Melanoma	Preclinical	−	^358^
IACS‐8803	Synthetic CDN				
MK‐1454	Synthetic CDN	Advanced solid tumors; Lymphomas	I (NCT03010176)	Completed	^353^, ^354^
E7766	Synthetic CDN	Advanced solid tumors; Lymphomas	I (NCT04144140)	Completed	^355^, ^356^, ^357^
SB11285	Synthetic CDN	Melanoma; Head and neck squamous cell carcinoma; Solid tumor	I (NCT04096638)	Recruiting	
BI1387446	Synthetic CDN	Advanced solid tumors	I (NCT04147234)	Active, not recruiting	
IMSA101		Solid tumor	I/II (NCT04020185)	Completed	
DMXAA	DMXAA	Breast cancer	III (NCT00662597)	Terminated	^359^, ^360^
		Melanoma			
		Neuroendocrine tumor			
		Adrenocortical carcinoma			
		Acute myelogenous leukemia			
		Glioma			
		Non‐small cell lung cancer			
CMA	DMXAA analogue	Macrophages	Preclinical	−	^362^
α‐Mangostin	DMXAA analogue	Monocyte‐derived macrophages	Preclinical	−	^363^
Low‐dose radiation	Indirect STING agonist	Colorectal cancer cells	Preclinical	−	^365^
Chronic cisplatin	Indirect STING agonist	Epithelial ovarian cancer	Preclinical		^367^
PARPi (Olaparib)	Indirect STING agonist	Gynecological, breast, pancreatic and non‐small cell lung cancer	I (NCT06130254)	Not yet recruiting	^368^
		Breast cancer	III (NCT06112379)	Recruiting	
		Ovarian cancer	III/IV (NCT05952453)	Not yet recruiting	
CHK1i (Prexasertib)	Indirect STING agonist	Small cell lung cancer	II (NCT02735980)	Completed	^369^
ABZI	Small molecule STING agonist	Colon cancer	Preclinical	−	^370^, ^371^
MSA‐2	Small molecule STING agonist	Colorectal cancer	Preclinical	−	^372^, ^373^
SR‐717	Small molecule STING agonist	Melanoma	Preclinical	−	^372^, ^373^
SNX281	Small molecule STING agonist	Advanced solid tumor; Lymphoma	I (NCT04609579)	Active, not recruiting	^375^, ^376^
TAK‐676	Small molecule STING agonist	Advanced solid tumors	I (NCT04420884)	Recruiting	
GSK3745417	Small molecule STING agonist	Leukemia	I (NCT05424380)	Not yet recruiting	
MK‐2118	Small molecule STING agonist	Advanced solid tumor; Lymphoma	I (NCT03249792)	Completed	

Abbreviations: ABZI, amidobenzimidazole.; c‐di‐GMP, cyclic guanosine diphosphate; CDN, cyclic dinucleotide; cGAMP, cyclic guanosine adenosine monophosphate; CHK1i, checkpoint kinase 1 inhibitor; CMA, chaperone‐mediated autophagy; DMXAA, 5, 6‐dimethylxanthone‐4‐acetic acid; PARPi, poly (adenosine diphosphate‐ribose) polymerase inhibitor.

#### 7.2.1 Natural CDN

Known natural CDNs include exogenous c‐di‐GMP, c‐di‐AMP, and 3′,3′‐cGAMP in bacteria, and endogenous 2′,3′‐cGAMP in mammalian cells.^343^ These natural CDNs are often used in antitumor research as agonists of the cGAS–STING signaling pathway. In in vitro studies, c‐di‐GMP was shown to inhibit the proliferation of basal human colon cancer cells and human cecal adenocarcinoma cells.^344^ In in vivo studies, intraperitoneal injection of high‐dose c‐di‐GMP directly activated Caspase‐3, triggering apoptosis of mouse breast cancer 4T1 cells, and inhibiting the proliferation of breast cancer cells. In Eμ‐TCL1 transgenic mice with chronic lymphocytic leukemia, intraperitoneal injection of 3′,3′‐cGAMP inhibited the growth of multiple myeloma in vivo, thus preventing the development of leukemia.^345^ Moreover, endogenous 2′,3′‐cGAMP mediated DC activation and T‐cell cross‐activation to inhibit tumorigenesis in CT26 colon adenocarcinoma mice.^346^ Additional studies revealed that an injection of 2′,3′‐cGAMP into tumors (i.t.) significantly inhibited tumor growth and improved survival rate in breast cancer (4T1 luc), squamous cell carcinoma (mSCC1), colon cancer (CT26), and melanoma (B16F10) mouse models.^347^ Moreover, 2′,3′‐cGAMP inhibited lung metastasis in a melanoma (B16F10) mouse model, suggesting that CD8^+^ T cells induced by cGAMP drive the systemic antitumor immune response to control local and distant tumor growth.^348^

Although natural CDNs have strong antitumor effects, they have shortcomings that cannot be ignored.^349^ First, CDN is easily degraded by enzymes in vivo. Second, the membrane penetration of natural CDNs is insufficient, and their ability to activate STING is consequently poor. Third, natural CDNs can cause side effects when used as a treatment therapy. Therefore, methods for administering CDNs should be improved or natural CDNs should be modified to improve their drug properties and reduce side effects.

#### 7.2.2 Synthetic CDN

Compared with natural CDNs, synthetic CDNs exhibit excellent performance. For example, the nonbridged oxygen atom in the phosphodiester bond of cGAMP is replaced by a sulfur atom to obtain the modified compound 2′,3′‐cGsAsMP, which shows resistance to the degradation of 2′,3′‐cGAMP hydrolase, yet still exhibits high affinity for human STING (*h*STING).^191^ ADU‐S100, another synthetic CDN, activates all *h*STING variants and mouse STING. Due to its enhanced stability and lipophilicity, ADU‐S100 activates STING signal transduction with higher efficiency than endogenous or exogenous CDNs, and it was proven to have a good tumor elimination effect in melanoma (B16), breast cancer (4 T1), colon cancer (CT26), and other mouse tumor models.^350^ However, ADU‐S100 results in clinical trials have been disappointing. One clinical trial is a Phase I study (NCT02675439) as a monotherapy in patients with advanced/metastatic cancer,^351^ and the other is a Phase II study in combination with anti‐PD1 pembrolizumab in patients with head and neck cancer (NCT03937141).^352^ The factors leading to the poor clinical effect of ADU‐S100 need further study. In addition to ADU‐S100, several other CDN‐based STING agonists have been designed. MK‐1454 exhibits high‐affinity for STING and downstream induced IFN‐β secretion is strong. In addition, intratumoral injection of MK‐1454 induced complete tumor regression.^353^ A Phase I clinical trial of MK‐1454, alone or in combination with Pembrolizumab, in patients with advanced solid tumors or lymphoma has been completed (NCT03010176). Preliminary data revealed that MK‐1454 was effective in combination with Pembrolizumab, although MK‐1454 was not effective as a monotherapy.^354^ E7766, another macrocyclic STING agonist, was demonstrated to exhibit strong activity, and intratumoral administration of E7766 resulted in tumor growth inhibition or regression in mouse models.^355^ A Phase I clinical trial of E7766 for solid tumors and lymphomas has recently been completed (NCT04144140), but the clinical results have not been disclosed. Other Synthetic CDNs, including SB11285, BI1387446, and IMSA101, are also undergoing clinical evaluation (NCT04096638, NCT04147234, and NCT04020185, respectively), although their clinical results have not yet been disclosed.^356^, ^357^ In addition, two highly effective 2′,3′‐thiophosphate CDN analogs, IACS‐8779 and IACS‐8803, have been developed. Using the melanoma (B16) mouse model, IACS‐8779 and IACS‐8803 were demonstrated to induce an extremely strong systemic antitumor response (in comparison with ADU‐S100 or cGAMP).^358^

In summary, synthetic CDNs as STING agonists exhibit better stability and antitumor activity (compared with natural CDNs), and many clinical trials of these compounds are ongoing or have been completed. However, therapeutic applications of synthetic CDNs are currently limited to the treatment of tumors, and other clinical applications of these synthetic CDNs have not been approved. Therefore, novel STING agonists suitable for whole‐body delivery should be developed through the modification and optimization of synthetic CDN structures.

#### 7.2.3 DMXAA and its analogs

DMXAA is a non‐CDN STING agonist. Intraperitoneal injection of DMXAA markedly inhibits the growth of breast cancer in rats.^359^ In addition, DMXAA has shown various antitumor effects in different mouse models, including in melanoma (B16), gastrointestinal and pancreatic neuroendocrine tumor, adrenocortical carcinoma, acute myeloid leukemia, glioma (GL261), and lung cancer mouse models.^360^ However, DMXAA has performed poorly in clinical trials because its interaction is limited to mSTING. A Phase III clinical trial of DMXAA in patients with advanced non‐small cell lung cancer (NSCLC) (NCT00662597) was stopped due to lack of efficacy.^361^ Another similar derivative called 10‐carboxymethyl‐9‐aridinone (CMA) was also identified as a specific agonist of mSTING.^362^ While the results of these trials are disappointing, DMXAA has theoretical value in the design of high‐affinity non‐nucleotide *h*STING agonists. Furthermore, additional research has revealed that the DMXAA derivative α‐Mangostin is more effective in activating *h*STING than mSTING.^363^ In conclusion, the application of rational design concepts to DMXAA analogs has contributed to the development of new tumor therapies.

#### 7.2.4 Indirect STING agonists

Several studies have found that classical anticancer therapies such as radiotherapy, chemotherapy, and targeted therapy may activate the cGAS–STING signaling pathway, thus providing an indirect STING agonist for tumor treatment.^364^ For example, irradiated tumor cells directly release genomic DNA fragments into the cytoplasm or shuttle their self‐DNA wrapped in the exosome into immune cells to initiate the STING‐dependent immune response.^364^ Moreover, low‐dose radiotherapy blocks the activation of TREX1 and stimulates the release of DNA fragments into the cytoplasm to activate the STING signaling pathway.^365^ Additionally, chemotherapy is known to induce DNA damage, which activates the cGAS–STING signal to promote DC‐mediated Ag presentation and T‐cell activation.^59^, ^366^ In a mouse model of epithelial ovarian cancer, chronic cisplatin treatment promoted the aggregation of T cells in tumors and the immunogenicity of advanced tumors through the cGAS–STING signaling pathway.^367^ It is worth noting that targeted therapy can also enhance the STING‐mediated immune response. For example, treatment of NSCLC cells with the poly (adenosine diphosphate‐ribose) polymerase inhibitor (PARPi) Olaparib produced cytoplasmic chromatin fragmentation similar to micronuclei that promote activation of the cGAS–STING signaling pathway and the production of downstream CCL5.^368^ In recent years, Olaparib has being investigated in phase I, phase III, and phase III/IV clinical trials (NCT06130254, NCT06112379, NCT05952453, respectively) for the treatment of gynecological, breast, pancreatic, and non‐small cell lung cancers (https://trialsearch.who.int/Trial2.aspx? TrialID=NCT06130254, NCT06112379, NCT05952453). In addition, Prexasertib, a checkpoint kinase 1 (CHK1) inhibitor (CHK1i), was demonstrated to induce DNA double‐strand breaks to activate STING signaling, thereby enhancing T‐cell aggregation in tumors in mice with small cell lung cancer (SCLC).^369^ A Phase II clinical trial of Prexasertib for the treatment of SCLC has been completed (NCT02735980) (https://trialsearch.who.int/Trial2.aspx? TrialID=NCT02735980), although the results have not yet been disclosed. In summary, STING signaling pathway is activated to mediate the immune response during traditional cancer treatment, and this may improve the overall clinical therapeutic effect. These results suggest that STING signaling pathway may be combined with other therapeutic methods to expand its clinical application.

#### 7.2.5 Small molecule STING agonists

A non‐CDN STING agonist called amidobenzimidazole (ABZI) has been reported to be 400 times more potent than cGAMP, and it induced dose‐dependent STING signal activation in PBMCs. In the colorectal tumor model (CT‐26) of BALB/c mice, ABZI treatment led to significant regression of tumors and a significantly improved survival rate. ABZI is the first effective non‐CDN STING agonist with systemic antitumor activity in model mice, and it is considered the most promising non‐nucleotide small molecule STING agonist.^370^, ^371^

MSA‐2 (benzothiophenoxybutyric acid), a bicyclic benzamide, is another promising non‐nucleotide STING agonist. This *h*STING‐specific agonist can be used as a chemical inducer of IFN‐β secretion.^372^, ^373^ Studies have shown that MSA‐2 achieved dose‐dependent antitumor activity when administered orally or subcutaneously in the MC38 homologous mouse tumor model. Notably, complete tumor regression and good drug tolerance were observed in 80−100% of treated mice, and significant increases in IFN‐β, IL‐6, and TNF‐α were observed in the tumor and plasma. MSA‐2 combined with antiprogrammed death receptor 1 (PD‐1) antibody in treating CT26, MC38, and B16F10 mouse tumors was superior to a single drug in inhibiting tumor growth and increasing the survival rate. In addition, MSA‐2 was demonstrated to more easily enter tumor cells in the TME (compared with normal tissues), which is conducive to specific inhibition of tumor cell growth.^372^, ^373^

The efficacy of SR‐717, a non‐nucleotide small molecule STING agonist administered to the whole body, was not affected by allelic or interspecific differences in the STING amino acid sequence. SR‐717 was subsequently demonstrated to exhibit strong tumor growth inhibition of highly aggressive B16F10 tumors. In addition, SR‐717 prolonged survival and was well tolerated. Compared with anti‐PD‐1 or anti‐programmed death ligand 1 (PD‐L1) monotherapy, intraperitoneal administration of SR‐717 was highly efficacious in the inhibition of tumor growth and in prolonging the overall survival rate.^373^, ^374^ Furthermore, SR‐717 also successfully prevented metastasis of melanoma to the lungs of mice, and it enhanced the activation of CD8^+^ T lymphocytes in the TME and lymph nodes.^373^, ^374^

In addition to the small molecule STING agonists mentioned above, several other small molecule STING agonists have been to clinical trial, including SNX281, TAK‐676, GSK3745417, and MK‐2118. SNX281 was demonstrated to induce THP‐1 cells and human PBMCs to secrete IFN‐β, TNF‐α, and IL‐6 in a STING‐dependent manner. Moreover, SNX281 exhibited significant antitumor activity in tumor models, including MC38, CT26, and B16‐F10, when used alone or in combination with anti‐PD‐1 antibodies.^375^ A Phase I clinical trial of SNX281 alone and in combination with pembrolizumab for advanced solid tumors and lymphomas is currently underway (NCT04609579) (https://trialsearch.who.int/Trial2.aspx? TrialID=NCT04609579). The STING agonists TAK‐676, GSK3745417, and MK‐2118 are also in Phase I clinical trials for monotherapy and in combination with anti‐PD‐1 antibodies for advanced cancer (NCT04420884, NCT05424380, and NCT03249792, respectively). However, the results of these clinical trial have not yet been disclosed (https://trialsearch.who.int/Trial2.aspx? TrialID=NCT04420884, NCT05424380, NCT03249792).

In summary, several small molecule STING agonists are still in preclinical or early clinical development. These STING agonists induce long‐lasting tumor regression, and they also work synergistically with immune checkpoint inhibitors against tumors. It is hoped that anticancer drugs with higher activity that are more suitable for systemic administration can be developed based on the above research on small molecule STING agonists.

### 7.3 Application of the cGAS–STING agonist in cancer immunotherapy

The application of cGAS–STING agonists in cancer model mice and clinical treatments has been described in a previous article. Here, we introduce progress in their combination with chemoradiotherapy, immune checkpoint blocking, cancer vaccine, and CAR‐T cells in cancer immunotherapy (Figure 7).^377^

#### 7.3.1 Combined with chemoradiotherapy

Radiotherapy and chemotherapy are mostly used for the treatment of solid tumors.^378^ However, radiation and chemical drugs can both induce micronucleus formation and the leakage of chromatin fragments into the cytoplasm. Research suggests that chemoradiotherapy activation of the cGAS–STING signaling pathway enhances the antitumor immune response. Thus, chemoradiotherapy combined with STING agonists should show enhanced antitumor effects, reduced toxicity, and fewer side effects. The immunity activated by STING agonists is a strong adjuvant effect that enhances the adaptive immune response to tumor Ags exposed during radiotherapy.^78^, ^83^ In a mouse model of pancreatic cancer, CT‐guided radiotherapy was combined with STING agonists to inhibit local and remote tumors synergistically. In addition, cGAMP combined with 5‐FU improved the antitumor activity of 5‐FU and reduce its toxicity.^379^

#### 7.3.2 Combined with immune checkpoint blocking therapy

Cytotoxic T lymphocyte Ag‐4 (CTLA‐4) and PD‐1 are important coinhibitory molecules that cause T lymphocyte dysfunction, ultimately promoting tumor cell evasion of host immune clearance. Therefore, antitumor therapies that antagonize CTLA‐4 and/or PD‐1/PD‐L1 can attenuate the immune escape signal released by the tumor and enhance the host antitumor immune response.^380^, ^381^, ^382^ However, there are not enough CD8^+^ T cells in the TME, and immune checkpoint inhibitors are difficult to functionalize.^383^, ^384^, ^385^ The injection of STING agonists into tumors promoted T‐cell aggregation in the TME by inducing the production of C‐C motif ligand 5 (CCL5) and C‐X‐C motif ligand 10 (CXCL10). Therefore, STING agonists are ideal sensitizers for anti‐PD‐1/PD‐L1 treatment of tumors.^372^, ^386^ The antitumor activity of anti‐CTLA‐4 and/or anti‐PD‐1 antibodies was significantly enhanced in melanomas through combination therapy with STING agonists. Moreover, anti‐PD‐1 antibody therapy exhibited a stronger antitumor effect in a mouse model of squamous cell carcinoma when combined with STING agonist (compared with antibody monotherapy).^374^, ^387^ Notably, phase I clinical trial results confirmed that intratumoral injection of the STING agonist MK‐1454 could enhance the antitumor effect of anti‐PD‐1 and eventually lead to tumor regression.^388^ Crucially, anti‐CTLA‐4 combined with STING agonist therapy failed to cause tumor regression in a mouse tumor model established with STING‐deficient B16 tumor cells.

#### 7.3.3 Combined with cancer vaccine therapy

STING agonists can be delivered together with tumor Ag peptides as vaccine adjuvants to enhance the antitumor immune response.^389^ For example, mice with metastatic breast cancer (4T1) were treated with an attenuated Listeria monocytogenes (LM) vaccine in combination with a low dose of c‐di‐GMP, resulting in almost complete elimination of metastatic tumors.^372^ Moreover, a STING agonist and granulocyte‐macrophage colony stimulating factor (GM‐CSF)‐secreting cancer vaccine (GM‐VAX) were combined to form STING‐VAX, which exhibited an antitumor effect in various cancer models, including melanoma, colon cancer, gastrointestinal squamous cell carcinoma, and pancreatic cancer. Mice treated with STING‐VAX also demonstrated a significant increase in CD8^+^ IFN‐γ^+^ T cell infiltration (compared with GM‐VAX without STING agonists). In addition, after dorsal transplantation of B16 melanoma in mice, contralateral injection of STING‐VAX significantly inhibited tumor growth in a dose‐dependent manner. Compared with GM‐VAX, STING‐VAX enhanced T‐cell infiltration in tumor tissue. Therefore, the antitumor effect of a cancer vaccine can be increased by combining it with cGAS–STING agonist treatment.^390^, ^391^

#### 7.3.4 Combined with CAR‐T cell therapy

Cancer immunotherapy using chimeric Ag receptor‐modified T cells (CAR‐T) has a good clinical effect on hematological malignancies, but there are still challenges in using CAR‐T‐cell therapy to treat solid tumors.^392^ A recent study revealed that STING agonists improved the effectiveness of CAR‐T‐cell therapy during the treatment of pancreatic cancer and melanoma, and codelivery of STING agonists and CAR‐T cells could effectively activate the immune response to eliminate tumor cells that were not recognized by adoptive metastatic lymphocytes. Therefore, combination treatment with STING agonists can also prevent the emergence of tumor cell escape mutations.^393^

### 7.4 cGAS–STING agonist delivery system

Because of the hydrophilicity of cGAS–STING agonists, their negative charge, and their lability to enzyme degradation, the antitumor immunity potential of these important therapeutics and their biological treatment effects are typically constrained. To optimize the potential of cGAS–STING agonists, effective drug delivery systems are essential. To date, many different cGAS–STING agonist delivery systems have been developed. Here, we introduce three widely studied cGAS–STING agonist delivery systems: nanocarrier, microparticle (MP), and hydrogel.^394^

#### 7.4.1 Nanocarrier

Liposome‐encapsulated STING agonists form an effective nanobiological drug. In melanoma‐bearing mice, injected liposomal cGAMP was absorbed by APCs and released into the cytoplasm, where it activated the cGAS–STING signaling pathway.^395^ In comparison with the delivery of soluble cGAMP alone, STING agonist‐dependent innate immune activation and associated antitumor activities were enhanced after delivery of liposome nanoparticles (NPs) coated with cGAMP (cGAMP‐NPs). Notably, in a PD‐L1‐insensitive triple‐negative breast cancer model, cGAMP‐NPs were demonstrated to induce the production of proinflammatory cytokines and IFN‐I to fight cancer. In addition, researchers have used polyethylene glycol (PEG) liposome carriers to replace soluble CDNs, largely because of the associated risk of systemic inflammatory toxicity. These PEG liposome carriers of CDNs were found to stably and effectively induce activation of APCs and to activate the T‐cell response. Therefore, use of a liposome delivery system coated with a STING agonist as a vaccine adjuvant can greatly improve the associated antitumor effect and immune response.^396^ It has also been reported that nanocarriers can enter and adapt to the immunosuppressed TME. In a mouse lung metastasis model, a proinflammatory reaction was observed at the tumor metastasis site when liposomal cGAMP was applied. In addition, liposomal cGAMP was demonstrated to enter the TME, control lung metastasis, and prolong survival time.^397^ In summary, in an immunosuppressive TME, liposomes coated with CDNs can effectively overcome the immunosuppressive effect of the TME and produce antitumor immunity.

Polymer nanocarriers can also be used to transport STING agonists. Studies have shown that polymer nanocarriers can increase cGAMP activity in monocytes, macrophages, and melanoma cell lines. Moreover, STING agonists in polymer nanocarriers have been reported to trigger a T‐cell‐mediated immune response in the TME. This was evidenced by increased levels of tumor‐infiltrating neutrophils, the polarization of M2 macrophages toward proinflammatory M1 macrophages, overexpression of CD86 in tumor‐draining lymph nodes, and increased numbers of CD8^+^ T cells.^398^ Separately, synthetic polymer NPs comprised of a polyvalent STING agonist (PC7A NPs) were demonstrated to induce strong Ag‐specific cytotoxic T lymphocyte (CTL), Th1, and Th2 responses. A mechanistic study revealed that PC7A NPs promoted Ag delivery and APC cross‐presentation to stimulate the CD8^+^ T cell response jointly. Importantly, in *Ifn‐α/β* ^−/−^ mice, it was verified that PC7A NPs were demonstrated to enhance the immune response through the cGAS–STING signaling pathway after activating APCs in draining lymph nodes.^399^ Recently, Xing et al.^400^ developed a multistimuli activatable peptide nanodrug, which achieved the site‐specific release of PD‐L1 antagonist peptide (CVR) to block the PD‐1/PD‐L1 pathway, while simultaneously activating the cGAS–STING pathway to initiate robust and durable T cell antitumor immunity. Finally, combination nanotherapeutics based on the activation of the cGAS–STING signaling pathway have been used in tumor targeting therapy, including emerging strategies combining nanoformulated agonists with chemotherapy, radiotherapy, as well as other immunomodulation methods.^401^

#### 7.4.2 Microparticle

MPs are widely used for activation induction based on cGAS–STING signaling pathways. For example, tumor cell‐derived particles (T‐MPs), membrane particles produced by apoptotic cancer cells, can be used as delivery vesicles. T‐MPs present DNA fragments from cancer cells to APCs and stimulate the production of IFN‐I by activating the cGAS–STING signaling pathway. Subsequently, IFN‐I induces DC maturation by upregulating CD80, CD86, and MHC‐II. The mature DCs then present tumor Ags to CD8^+^ T cells, triggering an antitumor immune response.^402^, ^403^ Another MP delivery system is Acid‐sensitive acetylated dextran (Ace‐DEX) polymer MPs. In comparison with soluble cGAMP, Ace‐DEX MPs encapsulated in cGAMP have no toxicity. Moreover, they increase the expression levels of IFN‐I 1000‐fold in vitro and 50‐fold in vivo (compared with soluble cGAMP).^404^ In a comparative study, Ace‐DEX MPs were coated with cGAMP, IMQ, morabutide, or poly (I:C). The results reveal that Ace‐DEX MPs containing cGAMP are the most effective drug for inhibiting tumor growth.^405^

#### 7.4.3 Hydrogel

Hydrogels are commonly used as carriers for the local release of antitumor drugs. In particular, crosslinked hyaluronic acid (HA) hydrogel scaffolds exhibit good biocompatibility and the ability to control drug release. Intraoperative injection of hydrogels containing STING agonists into tumors has demonstrated therapeutic benefits. Thus, the hydrogels containing STING agonists were observed to modulate the resection microenvironment by triggering immune cells.^406^, ^407^ Similarly, linear polyethyleneimine/HA hydrogel loaded with cGAMP was demonstrated to stimulate IFN‐β and IL‐6 production in macrophages.^408^ Matrigel is a thermally responsive hydrogel containing laminin and collagen IV. After combination with a STING agonist, the treated Matrigel was used to cure local tumors in the resected cavities of head and neck squamous cell carcinoma model mice.^409^ STINGel is the third peptide hydrogel used for intratumor delivery of CDNs, and this releases CDNs more slowly than ordinary collagen hydrogel. Moreover, STINGel can achieve sustained release of high concentrations of CDNs.^410^

In summary, many cGAS–STING signaling pathway inhibitors, agonists, cancer immunotherapy strategies, and agonist drug delivery systems have been reported in recent years. However, several problems must be overcome before the cGAS–STING signaling pathway can be productively exploited to yield clinical therapies. First, STING agonists are currently only available for intratumoral injection, and existing compounds should be actively modified or novel compounds screened for stable systemic administration. Second, it remains to be investigated whether the agonist‐activated cGAS–STING signal triggers a negative feedback effect when using targeted drugs. Third, individual differences should be taken into account during the drug development process, and safety and efficacy should both be considered to avoid excessive activation of STING, which may cause a severe inflammatory response, and potentially even a “cytokine storm.”

## 8 SUMMARY AND OUTLOOK

Since the discovery of the cGAS–STING signaling pathway in 2013, great progress has been made on the origin, function, and regulating mechanism of the cGAS–STING signaling pathway in the past decade. Studies have shown that cGAS–STING's immune function and signal transduction have an ancient evolutionary origin, and human cGAS–STING signal originates from ancient mechanisms of phage defense in bacteria. After continuous functional evolution, the cGAS–STING signaling pathway can be activated by a variety of DNAs from pathogenic microorganisms (viruses, bacteria, and parasites) and host self cells (abnormal cell genomes, mitochondria, and micronucleus). Signal transduction of cGAS–STING is complex and rigorous, and there are multiple modification mechanisms to promote or inhibit this signaling pathway, such as phosphorylation, ubiquitination, sumoylation, glutamylation, proteolysis, acetylation, and methylation. There is compelling evidence to support a strong link between cGAS–STING and inflammatory diseases and tumors, and the important roles of cGAS–STING have been accepted in the occurrence and development of diseases such as pathogenic microbial infections, cancers, autoimmune diseases, neurological diseases, and visceral inflammation. Based on the numerous upstream triggers, complex regulating mechanisms, and functions in multiple inflammatory diseases of the cGAS–STING signaling pathway, we conclude that cGAS–STING has a broad and important function, acting like a hub with a large communication network.

However, the triggering, transduction, and function of the cGAS–STING signaling pathway are still incompletely known and need to be further explored. (1) Previous studies have confirmed that DNAs from pathogens or the host itself are recognized by cGAS only when exposed to cytoplasm, but the length and conformation of DNA fragments cannot be strictly defined. It is suggested that the properties of these DNAs, activated the cGAS–STING signaling pathway, should be explored and defined in the future, which is of great significance for revealing the pathogenesis of human diseases. (2) There are numerous promoting and inhibiting effects in the process of cGAS–STING signal transduction, and how they are triggered has yet to be fully clarified. In addition, whether and how such a huge and complex regulatory network is crosstalk needs to be discovered. Future studies should involve the activation state and change rule of the cGAS–STING signal when two or more kinds of promoting and inhibiting effects exist simultaneously, which will help to explain the occurrence and development mechanism of inflammatory diseases and cancers. (3) In the pathogenesis of cancers, the cGAS–STING signaling pathway has two opposite functions: tumor suppression and tumor promotion. Although existing studies have preliminarily concluded that it may be attributed to different development stages or different types of cancers, how these effects are transformed is still unknown. In the future, the functional transformation mechanism of the cGAS–STING signaling pathway in cancers will continue to be further studied, which will provide theoretical support for the study of the pathogenesis of cancer and the therapeutic effect targeting cGAS–STING signaling pathway. (4) DNA‐mediated activation of cGAS–STING signal induces inflammatory responses, which is one cause of inflammatory diseases. This review summarized the function and mechanism of the cGAS–STING signaling pathway in relevant inflammatory diseases that have received relatively high attention. However, it will be a long‐term research focus to continuously explore the important function of the cGAS–STING signaling pathway in other diseases, which will expand the scope of action of the cGAS–STING signaling pathway and provide the theoretical basis for the development of broad‐spectrum drugs targeting cGAS–STING signaling pathway.

In recent years, more and more drugs targeting the cGAS–STING signaling pathway have been developed. For diseases with excessive or abnormal activation of the cGAS–STING signaling pathway, inhibitors of cGAS, STING, and TBK1 have been designed and have achieved good preclinical therapeutic results. Indeed, these inhibitors can effectively inhibit the activation of the cGAS–STING signaling pathway. However, long‐term inhibition of cGAS–STING signal activity may increase the risk of pathogenic microbial infection or cancer. Of concern, given the diversity of triggers, regulating mechanisms, and functions of the cGAS–STING signaling pathway, total inhibition of the cGAS–STING signal as a therapeutic approach may not be a viable strategy. Moreover, current agonists targeting cGAS–STING are mainly limited to cancer therapy, and systemic drug delivery has also not been achieved. The greatest risk is that uncontrolled STING agonists or inhibitors may impair normal physiological functions and produce serious side effects, which greatly limits their use in the treatment of tumors and autoimmune diseases. Therefore, it is required to determine the safe dose of STING agonists or inhibitors accurately. Furthermore, future studies on the cGAS–STING signaling pathway as a therapeutic target should focus on inhibiting the inflammatory response caused by pathological activation of the cGAS–STING signal, or activating cGAS–STING signaling pathway to induce antitumor immune response without affecting its normal physiological function. Simultaneously, for different key regulating factors of the cGAS–STING signaling pathway, the combination of multiple targets and multiple drugs is also attractive. In fact, the key is that drugs targeting the cGAS–STING signaling pathway need to be targeted or specific to cells or tissues, which requires further research and improvement on the drug itself or the delivery system. Although the drug development based on the cGAS–STING signaling pathway is still in its infancy and there are many difficulties, further research on the regulating mechanisms of the cGAS–STING signaling pathway and the development of targeted drugs still provide new strategies and hopes for the targeted therapy of inflammatory diseases and cancers.

## AUTHOR CONTRIBUTIONS

Qijie Li conceived, wrote, and edited the manuscript. Ka Li and Hongbo Hu edited and revised the manuscript. Ping Wu, Qiujing Du, and Ullah Hanif provided significant assistance. All authors have read and approved the final manuscript.

## ACKNOWLDGEMENTS

All figures were created by authors using the WPS office. This work supported by China Postdoctoral Science Foundation, Grant/Award Number: 2019M653413; National Natural Science Foundation of China, Grant/Award Number: U22A20334; Natural Science Foundation of Chengdu Medical College, Grant/Award Number: CYZ19‐38.

## CONFLICT OF INTEREST STATEMENT

The authors declare no conflict of interest.

## ETHICS STATEMENT

The authors declare that ethics approval was not needed for this study.

## Data Availability

Not applicable.
